# Origins of N_2_O Selectivity Limits in Catalyzed Ammonia Oxidation

**DOI:** 10.1021/acscatal.5c07065

**Published:** 2026-02-04

**Authors:** Ivan Surin, Evgenii V. Kondratenko, Javier Pérez-Ramírez

**Affiliations:** † Institute of Chemical and Bioengineering, Department of Chemistry and Applied Biosciences, ETH Zurich, Vladimir-Prelog-Weg 1, 8093 Zurich, Switzerland; ‡ Advanced Methods for Applied Catalysis, 28392 Leibniz-Institut für Katalyse, Albert Einstein-Str. 29a, 18059 Rostock, Germany; § NCCR Catalysis, 8093 Zurich, Switzerland

**Keywords:** ammonia oxidation, nitrous oxide, CeO_2_-based catalysts, kinetics, reaction network analysis

## Abstract

Ammonia (NH_3_) oxidation to nitrous oxide (N_2_O) is a promising route to obtain this selective oxidant, but controlling product distribution is inherently challenging because N_2_O occupies an intermediate nitrogen oxidation state between N_2_ and NO. Despite recent advances, leading CeO_2_-based catalytic systems have consistently encountered a selectivity limit in the range of 80–85%. Herein, CeO_2_-supported Mn single atoms are employed as a stable, selective benchmark to investigate the origins of the N_2_O selectivity losses. Thorough kinetic analysis revealed that direct oxidation of NH_3_ to N_2_ is the main reason for incomplete N_2_O selectivity. This reaction dominates in a thin upstream catalyst bed layer, driven by its strong dependence on the NH_3_ partial pressure that ensures dense surface coverage by N-containing intermediates and promotes their irreversible coupling to N_2_. However, due to the inhibiting effect of H_2_O, this reaction is increasingly hindered along the catalyst bed, with N_2_O becoming the dominant product. Based on these insights, N_2_O selectivity could be increased from 81% to 90% while N_2_ selectivity decreased to 6% by water cofeeding and adjusting reactant partial pressures to tune surface coverage by N-containing intermediates. Evaluation of side reactions revealed a negligible impact of N_2_O decomposition or N_2_O reduction on product distribution. Conversely, employing isotopic tracing, reduction of in situ-formed NO by NH_3_ was established as a significant route to secondary N_2_O, and to a lesser extent, N_2_. This was shown to be a general feature of CeO_2_-based catalysts, including Mn, Au, and Cr systems, providing a lever for selectivity control. This work demonstrates how kinetic analysis can disentangle complex reaction pathways and identify both catalyst- and process-level strategies to advance NH_3_ oxidation to N_2_O beyond current limits.

## Graphical abstract

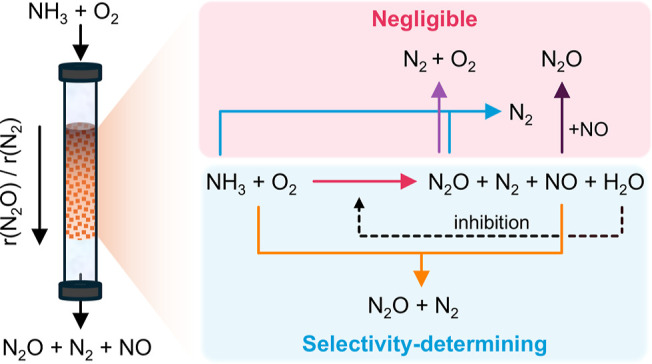

## Introduction

Ammonia (NH_3_) oxidation is of broad industrial relevance, yielding products from nitrogen N_2_ to nitrous oxide (N_2_O) and nitric oxide (NO). The latter is the main molecule of interest, being a crucial precursor for nitric acid production,^1^ while oxidation to N_2_ serves as a means of mitigating NH_3_ slip in internal combustion engines.^2,3^ N_2_O formation, in contrast, is typically regarded as an undesirable side reaction because of its environmental impact as a potent greenhouse gas.^4^ However, the unique potential of N_2_O as a selective oxidant for an array of hydrocarbon substrates, made possible by the ability to donate a single oxygen atom and suppress substrate overoxidation, has spurred interest in developing catalysts for N_2_O synthesis via NH_3_ oxidation.^5,6^

Selectivity control in NH_3_ oxidation is inherently challenging, as the N atom in N_2_O occupies an intermediate oxidation state between that in N_2_ and NO, making precise catalyst design crucial. Although N_2_ is the most thermodynamically stable product, the individual reactions to N_2_, N_2_O, and NO from NH_3_ are effectively irreversible under reaction conditions, meaning that high selectivity to either N_2_O or NO can be achieved under kinetic control, exemplified by NO production at very short contact times.^7^ Operating conditions add further complexity: low (<473 K) and high (>1073 K) temperatures promote N_2_ and NO formation, respectively, while optimal conditions for N_2_O fall within the broad intermediate window and strongly depend on the catalytic system. Feed composition, particularly the O_2_/NH_3_ ratio, also affects selectivity, though excess oxygen complicates separation and penalizes process economics.^8–10^ Considerable research efforts have therefore focused on catalyst design. Recent advances, particularly the use of redox-active CeO_2_ as a support, have enabled the selective N_2_O formation under stoichiometric conditions. Still, despite significant progress, all leading catalytic systems, namely CeO_2_-supported Au nanoparticles and single Mn atoms, as well as lattice-stabilized Cr atoms, have encountered a practical upper limit of 80–85% N_2_O selectivity.^11–13^ This underscores the need to understand the factors driving N_2_O formation and pinpoint the sources of the selectivity losses. Addressing this requires not only kinetic analysis of the primary NH_3_ oxidation pathways (eqs 1–3) but also consideration of secondary reactions involving oxidation products, such as N_2_O decomposition (eq 4), NO dimerization (eq 5) or NH_3_-mediated reduction of N_2_O (eqs 6 and 7) or NO (eqs 8 and 9), which have been observed in the context of NH_3_ oxidation to NO and N_2_.^14–17,19,20^ NO reduction by NH_3_, in particular, known as selective catalytic reduction (SCR), has been the subject of extensive research targeting N_2_ as an end product,^21^ but in the context of this work will also refer to the pathway leading to N_2_O (eq 10). The impact of these side reactions on targeted N_2_O synthesis remains unexplored, leaving the mechanistic picture incomplete and full selectivity elusive.^18^

1 $$4{NH}_{3}+3O_{2}\to 2N_{2}+6H_{2}O$$

2 $$4{NH}_{3}+4O_{2}\to 2N_{2}O+6H_{2}O$$

3 $$4{NH}_{3}+5O_{2}\to 4NO+6H_{2}O$$

4 $$2N_{2}O\to 2N_{2}+O_{2}$$

5 $$2NO\to N_{2}O+1/2O_{2}$$

6 $$2{NH}_{3}+3N_{2}O\to 4N_{2}+3H_{2}O$$

7 $$2{NH}_{3}+5N_{2}O\to 2NO+5N_{2}+3H_{2}O$$

8 $$4{NH}_{3}+4NO+O_{2}\to 4N_{2}+6H_{2}O$$

9 $$2{NH}_{3}+NO+{NO}_{2}\to 2N_{2}+3H_{2}O$$

10 $$4{NH}_{3}+4NO+3O_{2}\to 4N_{2}O+6H_{2}O$$

To address this issue, in this study, we undertook a comprehensive kinetic investigation in a spatially resolved manner and employing isotopically labeled ^15^NH_3_ to uncover the origins of N_2_O selectivity losses, using single manganese atoms supported on CeO_2_, Mn_SA_/CeO_2_, as the state-of-the-art system, combining high selectivity, stability, and a uniform active site structure. The catalyst was assessed in a fixed-bed reactor operated in the continuous flow mode at ambient pressure (Figure S1). First, the temperature dependence was evaluated to identify the optimal conditions for N_2_O synthesis. Subsequently, through variation of contact time, distinct selectivity-conversion trends were identified and kinetically evaluated to understand how the rates of formation of N_2_, N_2_O, and NO change along the catalyst bed. The spatially resolved kinetic analysis was extended by quantitatively assessing the effect of reactant and product partial pressures on the rates of formation of N_2_, N_2_O, and NO. To probe the role of secondary reactions involving the products of NH_3_ oxidation, namely, N_2_O decomposition and NH_3_-mediated reduction of N_2_O and NO, catalytic evaluation was conducted to determine the extent to which they could be contributing to observed product distribution. This was further complemented by isotopic tracing experiments with ^15^NH_3_ in NH_3_-mediated NO reduction, revealing distinct N_2_ and N_2_O formation pathways. We also extended this analysis to Au_NP_/CeO_2_ and CrCeO_*x*_ systems to assess whether the influence of side reactions is a general feature of CeO_2_-based NH_3_ oxidation catalysts. These findings identify possible reasons for the persistent selectivity gap and provide reaction- and catalyst-engineering strategies to mitigate these in the rational development of a catalytic process for the selective oxidation of NH_3_ to N_2_O.

## Experimental Methods

### Catalyst Synthesis

Cerium oxide, CeO_2_, was synthesized via the thermal decomposition of Ce­(NO_3_)_3_·6H_2_O (Sigma-Aldrich, 99%) in static air at 873 K (heating rate = 3 K min^–1^, hold time = 5 h). The resulting solid was ground into a powder in a mortar.

The Mn_SA_/CeO_2_ catalyst was synthesized by an incipient wetness impregnation method with a nominal metal content of 1 wt %. Accordingly, Mn­(NO_3_)_2_·4H_2_O (Alfa Aesar, 98%) was dissolved in deionized water, and the obtained solution was added dropwise to CeO_2_ prepared by thermal decomposition, as detailed above. After impregnation, the sample was dried under a vacuum at 353 K overnight and then calcined in static air at 823 K (heating rate = 3 K min^–1^, hold time = 5 h).

The Au_NP_/CeO_2_ catalyst was synthesized via deposition precipitation with urea with a nominal metal content of 1 wt %. Accordingly, CeO_2_ (Sigma-Aldrich, nanopowder <25 nm), the metal precursor HAuCl_4_·*x*H_2_O (ABCR, 99.9%, 49.5 wt % Au), and urea (Sigma-Aldrich, >99%) were added to deionized water. The suspension was magnetically stirred for 6 h at 353 K. The flask was covered with aluminum foil to prevent the potential photoreduction of Au. The suspension was then aged overnight, filtered, and washed with deionized water (1 L g^–1^). The solid was subsequently collected, dried overnight under vacuum at 353 K, and calcined in flowing air at 623 K (heating rate = 3 K min^–1^, hold time = 5 h).

The CrCeO_*x*_ catalyst was synthesized via the coprecipitation method with a nominal Cr content of 1 wt %. Accordingly, the metal precursors, Cr­(NO_3_)_3_·9H_2_O (Sigma-Aldrich, 99%) and Ce­(NO_3_)_3_·6H_2_O (Sigma-Aldrich, 99%), were dissolved in 80 cm^3^ water. H_2_O_2_ (Sigma-Aldrich, 50 wt % in H_2_O) was added to oxidize the Ce^3+^ to the more easily hydrolyzable Ce^4+^ with a molar ratio *n*(H_2_O_2_)/(*n*(Ce) + *n*(Cr)) = 3, as reported elsewhere.^12,22^ NH_3_ (VWR Chemicals, 25%) was added dropwise under vigorous stirring until a pH of 9.5 was reached. The suspension was further stirred for 24 h and subsequently filtered. The residue was washed with at least 1 L of water and dried overnight in a vacuum oven at 353 K. The dried powder was calcined in static air at 673 K (heating rate = 3 K min^–1^, hold time = 5 h).

### Catalyst Evaluation

Evaluation of catalytic performance in continuous-flow NH_3_ oxidation was conducted in a fixed-bed microreactor (Figure S1). All experiments were conducted at atmospheric pressure. The flow rate of gases, He (PanGas, purity 4.6, diluent), NH_3_ (PanGas, purity 3.8), ^15^NH_3_ (Eurisotop, 99.9% ^15^N), O_2_ (PanGas, purity 5.0), and Ar (PanGas, purity 5.0, internal standard), was regulated using thermal mass-flow controllers (Bronkhorst), connected to a mixing unit equipped with a pressure gauge. The catalyst (*m*_cat_ = 0.001–0.2 g; *d*_p_ = 0.1–0.4 mm; for tests at elevated gas-hourly space velocity, GHSV > 15,000 cm^3^ h^–1^ g_cat_^–1^, the catalyst bed was diluted with silicon carbide (*d*_p_ = 0.2–0.4 mm) to avoid the formation of hot spots) was loaded into a quartz microreactor (*d* = 8 mm or 2 mm), containing a bed made of quartz wool and placed in an electrical oven. The temperature in the middle of the catalyst bed was monitored and controlled by using a K-type thermocouple placed in a coaxial quartz thermowell. Prior to testing, the catalyst was heated in a He flow (*T*_bed_ = 473 K, *F*_T_ = 50 cm^3^ min^–1^) for 30 min, subsequently heated to the desired temperature, and allowed to stabilize for at least 30 min. For the evaluation of catalytic performance, the reaction mixture was fed at a total volumetric flow of *F*_T_ = 50–150 cm^3^ min^–1^.

Nitrogen-containing compounds (NH_3_, N_2_, N_2_O, NO_2_, and NO), as well as O_2_ and Ar were quantified via an online gas chromatograph equipped with a GS CP-Volamine column coupled to a mass spectrometer (GC-MS, Agilent, GC 7890B, MSD 5977A). Upon acquisition of the full chromatogram, individual ion chromatograms at *m*/*z* 17, 28, 30, 32, 40, 44, and 46 were extracted. A single peak was observed on chromatograms at *m*/*z* 32, 40, and 44, allowing one to directly quantify O_2_, Ar, and N_2_O, respectively. Sufficiently different retention times of N_2_O (*t* = 1.966 s), N_2_ (*t* = 1.887 s) and NO (*t* = 1.892 s) allowed resolution of the peaks attributed to N_2_O fragments, N_2_ and NO in the product stream at *m*/*z* 28 and 30 so that subsequent quantification of N_2_ and NO could be performed. Sufficiently different retention times of NH_3_ (*t* = 1.985 s) and H_2_O (*t* = 2.065 s) allowed the resolution of their respective peaks at *m*/*z* 17 and quantification of NH_3_.

The conversion of NH_3_ and O_2_ was calculated according to eq 11

11 $$X_{i}=\frac{{ṅ}_{i}^{in}-{\dot{\;n}}_{i}^{out}}{{ṅ}_{i}^{in}}$$

where *ṅ*_*i*_^in^ and *ṅ*_*i*_^out^ denote the molar flows of NH_3_ or O_2_ at the reactor inlet and outlet, respectively. Selectivity toward individual products was determined according to eq 12

12 $$S_{i}=\frac{\nu_{i}\cdot {ṅ}_{i}^{out}}{{ṅ}_{{NH}_{3}}^{in}\cdot X_{{NH}_{3}}}$$

where ν_i_ is the number of N atoms in the product molecule (i.e., ν = 2 for N_2_O or N_2_ and ν = 1 for NO). Nitrogen (*B*_N_) and oxygen (*B*_O_) balances were evaluated for each catalytic test according to eqs 13 and 14

13 $$B_{N}\left(\%\right)=\frac{{ṅ}_{{NH}_{3}}^{out}+2{ṅ}_{N_{2}O}^{out}+2{ṅ}_{N_{2}}^{out}+{ṅ}_{NO}^{out}}{{ṅ}_{{NH}_{3}}^{in}}\cdot 100$$

14 $$B_{O}\left(\%\right)=\frac{{ṅ}_{O_{2}}^{out}+2{ṅ}_{N_{2}O}^{out}+1.5{ṅ}_{N_{2}}^{out}+1.25{ṅ}_{NO}^{out}}{{ṅ}_{O_{2}}^{in}}\cdot 100$$

The error of *B*_O_ was less than 5% in all experiments. After the tests, the reactor was quenched to room temperature in He flow, and the catalytic materials were retrieved for further characterization.

### Assessment of Mass and Heat Transfer Limitations

The Carberry criterion (Ca)^23^ was used to evaluate external mass transfer limitations according to eq 15

15 $$Ca=\frac{r_{v,obs}}{a’\cdot k_{f}\cdot c_{b}}<\frac{0.05}{\left|n\right|}$$

where *c*_b_ is the bulk concentration of NH_3_ (1.45 mol m^–3^), *n is* the reaction order, and *r*_v,obs_, *a*’, and *k*_f_ denote the reaction rate, the specific particle area, and the mass transfer coefficient, respectively, which are derived via eqs 16, 17, and 18, respectively

16 $$r_{v,obs}=\frac{{ṅ}_{{NH}_{3}}^{in}-{\dot{\;n}}_{{NH}_{3}}^{out}}{V_{catalyst}}$$

17 $$a’=\frac{1}{L}=\frac{A_{p}}{V_{p}}=\frac{6}{d_{p}}$$

18 $$\frac{k_{f}\cdot d_{p}}{D}=1+{\left(1+\frac{v_{0}\cdot d_{p}}{D}\right)}^{1/3}$$

where *D* is the molecular diffusion coefficient of NH_3_ in He (7.58 × 10^–4^ m^2^ s^–1^), *L* is the characteristic length, *d*_p_ is the particle diameter, and *v*_0_ is the superficial fluid velocity. To assess nonisothermal extraparticle limitations,^23^ eq 19 was applied

19 $$\left|\gamma \cdot \beta_{e}\cdot Ca\right|=\left|\frac{E_{a}}{R\cdot T_{b}}\frac{\left(-\Delta H_{r}\right)\cdot k_{f}\cdot c_{b}}{h\cdot T_{b}}\cdot \frac{r_{v,obs}}{a’\cdot k_{f}\cdot c_{b}}\right|<0.05$$

where β_e_ denotes the external Prater number, γ is the dimensionless activation energy, *R* (8.314 J mol^–1^ K^–1^) is the universal gas constant, *E*_a_ is the activation energy, determined using Arrhenius eq (97 kJ mol^–1^), *T*_b_ is the temperature in the bulk material (673 K), Δ*H*_r_ is the reaction enthalpy (−1102.7 kJ mol^–1^), and *h* is the heat transfer coefficient (estimated at 100 J K^–1^ s^–1^ m^–2^).

Nonisothermal intraparticle limitations were evaluated using eq 20 ^24^

20 $$\left|\gamma \cdot \beta_{i}\cdot \Phi \right|=\left|\frac{E_{a}}{R\cdot T_{b}}\frac{\left(-\Delta H_{r}\right)\cdot D_{eff}\cdot c_{s}}{\lambda_{eff}\cdot T_{s}}\cdot \frac{r_{v,obs}\cdot L^{2}}{D_{eff}\cdot c_{s}}\right|<0.1$$

where β_i_ is the internal Prater number, Φ is the Wheeler–Weisz modulus, *c*_s_ is the surface concentration (*c*_s_ ≈ *c*_b_ in the absence of external mass transfer limitations), *T*_s_ is the temperature in the bulk material (673 K), λ_eff_ is the effective thermal conductivity (estimated at 0.5 W K^–1^ min^–1^), and *D*_eff_ is the effective diffusion coefficient, which can be derived via eq 21

21 $$D_{eff}=\frac{\varepsilon }{\tau }\cdot \mathrm{D̅}={\left(\frac{1}{D_{{NH}_{3},He}}+\frac{1}{D_{O_{2},He}}+\frac{1}{D_{K_{i}}}\right)}^{-1}$$

where τ is the tortuosity factor (estimated at 3), ε is the particle porosity (estimated at 0.2), *D*_NH_3_,He_ and *D*_O_2_,He_,_He_ are the molecular diffusion coefficients (7.58 × 10^–4^ m^2^ s^–1^ and 7.03 × 10^–4^ m^2^ s^–1^, respectively), and *D*_K_*i*__ is the Knudsen diffusion coefficient, which is calculated for components *i* (*i* = He, NH_3_, O_2_) in a cylindrical pore according to eq 22

22 $$D_{K_{i}}=97\cdot r_{pore}\cdot \sqrt{\frac{T}{M_{i}}}$$

where *r*_pore_ denotes the pore radius, *M*_*i*_ the molecular weight of component *i*, and *T* the temperature (673 K).

The results for selected conditions are given in Table S1. For all catalytic tests, the criteria for the absence of extra and intraparticle mass and heat transfer limitations are fulfilled, and therefore, the observed rate in all conducted catalytic tests was governed by intrinsic kinetics.

### Spatially-Resolved Steady-State Kinetic Analysis of Product Formation

Spatially resolved kinetic analysis was performed through assessment of steady-state catalytic performance in 9 different tests, with distinct catalyst amounts (*m* ^*i*^, *i* = 1–9, *m*_cat_ = 0.001–0.2 g) but the same total flow rate (150 cm^3^ min^–1^) and feed composition (8 vol % NH_3_, 8 vol % O_2_, 4 vol % Ar, and 20 vol % He), with the corresponding gas-hourly space velocity (GHSV) ranging from 15,000 cm^3^ h^–1^ g_cat_^–1^ to 9,000,000 cm^3^ h^–1^ g_cat_^–1^. The longest catalyst bed (*m* ^9^, 0.2 g) was divided into 9 sections. Section *i* corresponds to the space occupied by *m* ^*i*^ of the catalyst. These sections are termed the catalyst segments. To calculate the rate of NH_3_ consumption and product formation in each segment, i.e., segmental rates, we used the molar flow of NH_3_, *ṅ*_NH3_^out,*i*^, or product *j* (*j* = N_2_, N_2_O, NO), *ṅ*_*j*_^out,*i*^, from segment *i*, obtained in steady-state tests with the different *m* ^*i*^. The segmental rates of NH_3_ consumption and product formation were calculated according to eqs 23 and 24.

23 $$r_{{NH}_{3}}^{i}=\frac{{ṅ}_{{NH}_{3}}^{out,i}-{\dot{\;n}}_{{NH}_{3}}^{out,i+1}}{m^{i+1}-{\;m}^{i}}$$

24 $$r_{j}^{i}=\frac{{ṅ}_{j}^{out,i+1}-{\dot{\;n}}_{j}^{out,i}}{m^{i+1}-{\;m}^{i}}$$

## Results and Discussion

### CeO_2_-Based Catalysts for NH_3_ Oxidation

Recently, a range of high-performance catalytic systems for NH_3_ oxidation to N_2_O has been developed. These include Au nanoparticles (2–3 nm) on CeO_2_, Au_NP_/CeO_2_,^12^ isolated Cr sites incorporated into the lattice of CeO_2_, CrCeO_*x*_,^13^ and single Mn atoms supported on the surface of CeO_2_, Mn_SA_/CeO_2_.^11^ Despite differences in composition, these catalysts share common features: low metal content (1 wt %), moderate surface area (40–80 m^2^ g^–1^), and reliance on the redox activity of CeO_2_, which functions not only as a support but also as a cocatalyst by facilitating oxygen transport for the reaction following a Mars-van Krevelen-type mechanism. Together, these attributes enable activities surpassing previously reported catalysts and high N_2_O selectivity under stoichiometric feed conditions, consistently reaching values in the range of 80–85%. Among them, Mn_SA_/CeO_2_ was chosen as the benchmark for detailed kinetic investigation, as it has been extensively studied and characterized,^11^ proving to have a unique combination of high activity, selectivity, stability, and uniformity, as well as a well-defined single-atom structure, making it particularly well-suited for disentangling the mechanistic origins of N_2_O selectivity losses.

### Selectivity Trends

Temperature exerts a strong influence on product selectivity in NH_3_ oxidation, and the corresponding dependence was therefore first examined. The behavior of Mn_SA_/CeO_2_ follows the general trend of NH_3_ oxidation catalysts (Figure 1a). Namely, N_2_ formation dominates at low temperature and steadily drops as temperature increases, while N_2_O selectivity has a volcano-like shape, reaching a maximum N_2_O selectivity of 81% at 673 K before declining. NO formation remains low across the relevant range but gradually increases with temperature, consistent with expectations. It should be noted that at the applied GHSV, complete conversion of NH_3_ is already achieved at 573 K. To probe how selectivity changes with NH_3_ conversion, GHSV was varied at the temperature of maximum N_2_O selectivity (673 K; Figure 1b). A clear and pronounced effect of reducing the contact time on product selectivity was observed. As NH_3_ conversion decreased from 100 to 8%, N_2_O selectivity dropped almost linearly from 81 to 36%, which, in turn, was accompanied by a rise in N_2_ selectivity from 16 to 56%. At the same time, a less steep but steady increase in the NO selectivity from 2 to 8% was observed. The pronounced selectivity switch with increasing NH_3_ conversion is typically indicative of a reaction in series and would suggest that N_2_ is transformed into N_2_O if sufficient time is allowed. However, given the thermodynamic stability of N_2_, the possibility of such a reaction can be ruled out. Furthermore, by extrapolating the profiles to 0% NH_3_ conversion, we can see that the selectivity of all products is nonzero (62% N_2_, 28% N_2_O, and 10% NO), indicating that at least a portion of each product forms via the primary pathway, i.e., directly through oxidation of NH_3_.

**Figure 1:**
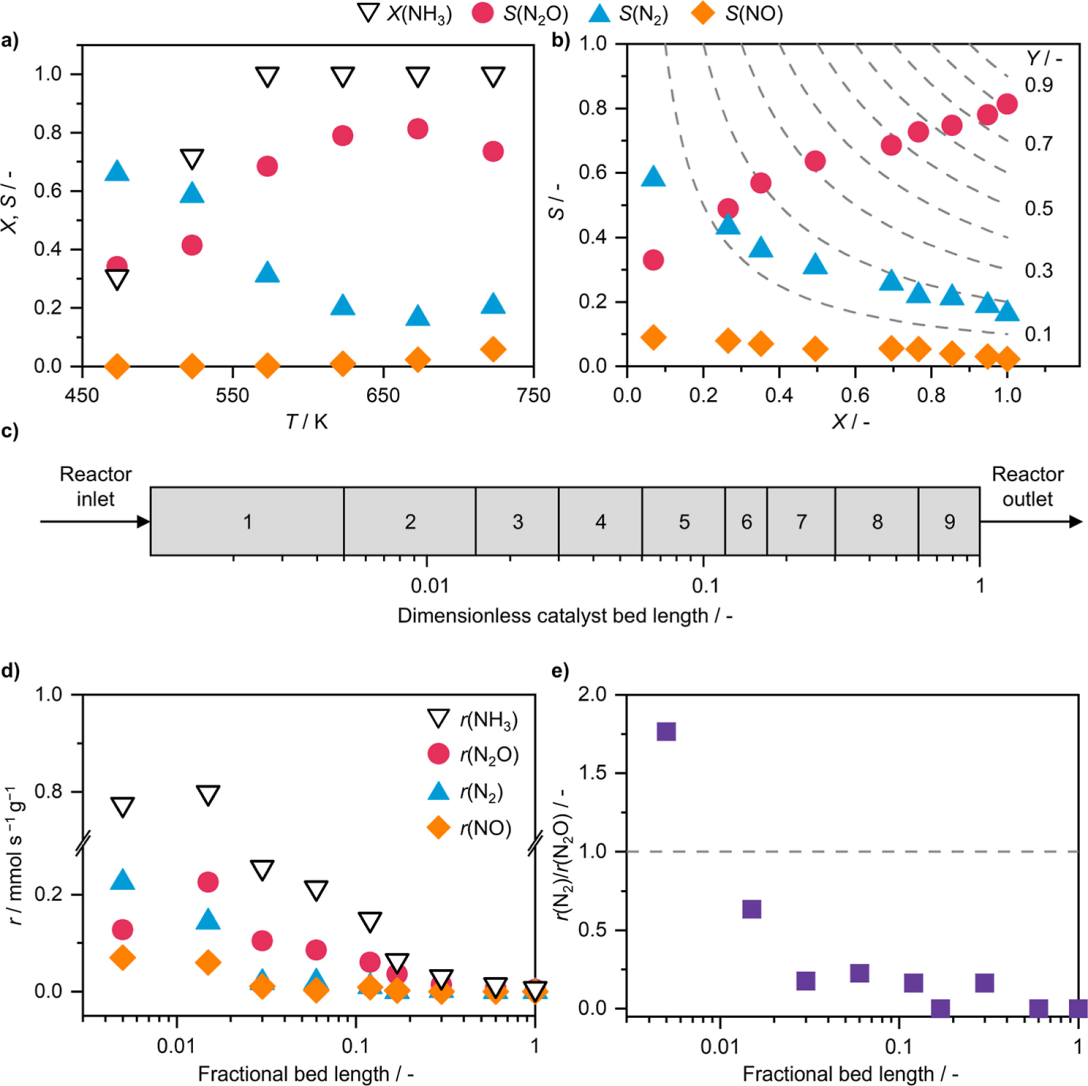
NH_3_ conversion and product selectivity of Mn_SA_/CeO_2_ in NH_3_ oxidation as a function of (a) reaction temperature and (b) NH_3_ conversion. (c) Schematic representation of the segments of the catalyst bed. (d) Segmental rates of NH_3_ consumption and product formation, *r*, and (e) ratio of the rates of N_2_ and N_2_O formation at different stages of the catalyst bed. Dashed lines in (b) represent product yield, *Y*. Reaction conditions: *T*_bed_ = (a) 473–723 K or (b,d–e) 673 K; *m*_cat_ = 0.001–0.2 g; GHSV = 15,000–9,000,000 cm^3^ h^–1^ g_cat_^–1^; feed = 8 vol % NH_3_, 8 vol % O_2_, 4 vol % Ar, He rest; *P* = 1 bar.

To rationalize the selectivity-conversion trends in Figure 1b, segmental rates of product formation and NH_3_ consumption along the catalyst bed were calculated (eqs 23 and 24, Figure 1c,d).^25,26^ It revealed that N_2_ formation dominates at the very early stages of the catalyst bed (∼1% of the total length) but quickly gets overtaken by N_2_O formation, which then continues to dominate across the remaining bed length (Figure 1e). The rate of N_2_ formation sharply declines within the first 10% of the catalyst bed and is practically zero in the remaining downstream catalyst layers. This trend is also valid for the rate of NO formation. The close-to-zero rates cannot be explained solely by the transition from differential to integral reactor operation because the rate of N_2_O formation is not zero. Thus, the formation of N_2_ and NO can be inhibited by reaction product(s) along the catalyst bed or affected by the secondary reactions of these products (vide infra). In contrast to N_2_ and NO, the rate of N_2_O formation increases in the first 2% of the catalyst bed and then decreases in the following layers. The rate of NH_3_ consumption also has a maximum in the same region as the rate of N_2_O formation. These spatial profiles suggest that, in addition to the primary NH_3_ oxidation to N_2_O, this product also arises from a secondary route involving NH_3_ and NO.

### Kinetics of NH_3_ Oxidation

#### Assessment of Mass- and Heat-Transfer Limitations

To rationalize the selectivity-conversion profiles in Figure 1b, it must first be established that the observed behavior arises from intrinsic kinetics and not as a consequence of adverse mass- and heat-transfer effects. The absence of internal mass transfer limitations was verified experimentally. Varying the size of catalyst particles from 0.4 mm down to powder of <0.1 mm had no appreciable effect on catalytic performance (Figure S2a), indicating that intraparticle mass-transport limitations are negligible. Similarly, varying the applied flow rate while maintaining a constant GHSV did not alter NH_3_ conversion or product distribution (Figure S2b), excluding extra-particle mass transport constraints. Heat transfer limitations were experimentally assessed in a series of catalytic tests (Figure S3), in which the same amount of catalyst was used but diluted with different amounts of SiC, an inert diluent with high thermal conductivity. No significant changes in the determined NH_3_ conversion or product selectivity were observed, indicating that neither significant hot spots nor heat-transfer limitations influence the measured reaction rates. This statement was further supported by evaluation of theoretical criteria (Table S1).^27^ Accordingly, using the data acquired at the highest and lowest applied GHSV in Figure 1b, the criteria for both extraparticle and intraparticle nonisothermal mass and heat transfer were satisfied. Thus, the measured reaction rates can be attributed to the intrinsic kinetics.

#### Temperature Dependence of Reaction Rates

Arrhenius analysis of the rates of NH_3_ consumption and product formation yielded apparent activation energies (*E*_a,app_) of 97 kJ mol^–1^ for NH_3_, 95 kJ mol^–1^ for N_2_O, 102 kJ mol^–1^ for N_2_, and 77 kJ mol^–1^ for NO (Figure S4). While these values themselves offer limited mechanistic information, their relatively large values are in the similar range as those in previous reports on comparable catalytic systems for NH_3_ oxidation^28,29^ and further confirm that the rate of reactions is governed by intrinsic kinetics and not transport phenomena. Accordingly, to gain deeper insight into the origin of observed selectivity trends, an examination of how the partial pressures of reactants and products affect the reaction rates is required.

#### Dependence of Reaction Rates on Reactant Partial Pressure

Varying the partial pressure of individual reactants, the effect on the overall rate of NH_3_ oxidation and the rate of formation of N_2_, N_2_O, and NO was monitored (Figure S5 and Table 1). All products displayed a positive dependence on ammonia partial pressure, (*p*(NH_3_)), though with notable differences in apparent reaction orders, *n*(NH_3_). For N_2_O and NO, the *n*(NH_3_) values are almost identical (0.34 and 0.36, respectively), whereas the value for N_2_ is nearly twice as high (0.66). While these noninteger values cannot be directly assigned to reaction stoichiometry, as they reflect the balance of surface coverages by reactive intermediates, the observed differences can nevertheless be rationalized. Namely, as NH_3_ adsorption on CeO_2_ is well-known to happen in a facile and abundant fashion,^30,31^ increasing *p*(NH_3_) leads to increased surface coverage by NH_*x*_ (*x* = 0–2) species generated via dehydrogenation of NH_3_ (eqs 25–27), while surface coverage by active lattice/adsorbed oxygen species is not affected, and the probability of two NH_*x*_ fragments coupling to N_2_ rises.

25 $${{NH}_{3}}^{*}+O^{*}\to {{NH}_{2}}^{*}+{OH}^{*}$$

26 $${{NH}_{2}}^{*}+O^{*}\to {NH}^{*}+{OH}^{*}$$

27 $${NH}^{*}+O^{*}\to N^{*}+{OH}^{*}$$

**Table 1:** Summary of Reaction Orders with Respect to Reactants and Products in Ammonia Oxidation, Shown for Both the Overall NH_3_ Conversion Rate and the Formation Rates of Individual Products

	Reaction order					
Reaction rate	*n*(NH_3_)	*n*(O_2_)	*n*(H_2_O)	*n*(N_2_O)	*n*(N_2_)	*n*(NO)
*r*(NH_3_)	0.51	0.56	–0.89	–0.09	–0.15	0.24
*r*(N_2_O)	0.34	0.66	–0.86	–0.07	–0.19	0.26
*r*(N_2_)	0.66	0.50	–0.95	–0.11	–0.11	0.49
*r*(NO)	0.36	0.41	–0.64	–0.07	–0.11	-

This can proceed via (i) direct coupling of N* species (eq 28), (ii) the pathway involving a hydrazine-like intermediate (N_2_H_4_*, eqs 29 and 30), or (iii) the imide route, wherein an imide (NH*) fragment combines with a nitroxyl (HNO*) intermediate, formed via oxidation of NH* (eqs 31 and 32).

28 $$N^{*}+N^{*}\to {N_{2}}^{*}+*$$

29 $${{NH}_{2}}^{*}+{{NH}_{2}}^{*}\to N_{2}{H_{4}}^{*}+*$$

30 $$N_{2}{H_{4}}^{*}+2O^{*}\to {N_{2}}^{*}+2H_{2}O^{*}$$

31 $${NH}^{*}+O^{*}\to {HNO}^{*}+*$$

32 $${HNO}^{*}+{NH}^{*}\to {N_{2}}^{*}+H_{2}O^{*}$$

The latter two have long been proposed to be among the main mechanisms of N_2_ formation in NH_3_ oxidation.^28,32^ Conversely, while N_2_O synthesis also requires that an N–N bond is formed, it is generally postulated to occur between HNO* intermediates (eq 33).^33,34^

33 $${HNO}^{*}+{HNO}^{*}\to N_{2}O^{*}+H_{2}O^{*}$$

Consequently, as higher *p*(NH_3_) drives greater consumption of lattice or adsorbed oxygen in NH_3_ dehydrogenation and increases the concentration of NH_*x*_, the probability of generating oxidized nitrogen intermediates diminishes relative to direct NH_*x*_–NH_*x*_ recombination. At the same time, the formation of NO involves only a single N-containing species, proceeding through oxidation of either a fully dehydrogenated N* species (eq 34)^35^ or of a HNO* intermediate (eq 35).^7,36^

34 $$N^{*}+O^{*}\to {NO}^{*}+*$$

35 $${HNO}^{*}+O^{*}\to {NO}^{*}+{HO}^{*}$$

This explains why the rate of N_2_ formation increases more strongly with *p*(NH_3_) than that of N_2_O or NO.

When considering the reaction orders with respect to O_2_, *n*(O_2_), we similarly observe a variation in the obtained values, albeit in a narrower range and following a distinct trend. Contrary to expectations, the *n*(O_2_) values do not increase with a degree of nitrogen oxidation. In fact, the rate of NO formation is least affected by *p*(O_2_), while the rate of N_2_O formation is affected the most, with N_2_ occupying an intermediate position. This behavior can be interpreted on the basis of considerations similar to those applied for *n*(NH_3_). Molecular oxygen can either be directly activated on the surface or be employed in vacancy healing within the CeO_2_ lattice. Two key roles of O-species in the reaction are (i) NH_3_ dehydrogenation (eqs 25–27 and 30) and (ii) oxidation of partially dehydrogenated NH_*x*_ fragments (eqs 30, 31, 34, and 35). The former is essential for all products and requires a higher stoichiometric input of O atoms, whereas the latter is specifically required for N_2_O and NO formation. Since only a single NH_3_ molecule needs to be dehydrogenated, followed by an oxidation to produce NO (1.25 mol O_2_ per mol of NO), N_2_ requires two NH_3_ to be dehydrogenated (1.5 mol O_2_ per mol of N_2_), while N_2_O requires both dehydrogenation and oxidation of two NH_3_ molecules (2 mol O_2_ per mol of N_2_O). The apparent oxygen dependence is the weakest for NO and progressively stronger for N_2_ and N_2_O.

#### Dependence of Reaction Rates on Product Partial Pressure

To assess whether product inhibition contributes to the observed selectivity trend, reaction orders with respect to oxidation products, including H_2_O, were also determined (Table 1, Figures S6 and S7). *n*(H_2_O) assumes a negative value for NH_3_ consumption and the formation of all products, confirming that water has a strong inhibiting effect. This is likely due to its competitive adsorption on the sites involved in the activation of the O_2_ to yield surface O* species required for NH_3_ dehydrogenation and concurrent H_2_O formation. Among the products, inhibition is strongest for N_2_ (−0.95), slightly weaker for N_2_O (−0.86), and weakest for NO (−0.64). Conversely, the strong H_2_O-mediated inhibition of N_2_ formation likely explains the practically zero rate of N_2_ in 90% of the downstream catalyst layers, as the H_2_O concentration progressively increases (Figure 1d). To probe this further, NH_3_ oxidation tests with cofed H_2_O were conducted (Figure S8). These confirmed that N_2_ formation is the most strongly suppressed at all conversions. At full NH_3_ conversion, H_2_O cofeeding increased N_2_O selectivity from 81% (no cofed H_2_O) to 84% and 85% with 12% and 36% H_2_O in the feed, respectively. A similar effect was observed for intermediate NH_3_ conversion (65%). At very low NH_3_ conversion (<10%), however, N_2_O selectivity decreased with H_2_O cofeeding, though the drop in N_2_ selectivity was even more pronounced. Specifically, the *r*(N_2_)/*r*(N_2_O) rate ratio decreased from 1.77 (no cofed H_2_O) to 1.5 (with cofed H_2_O), indicating stronger suppression of N_2_ formation. Meanwhile, both the formation rate and selectivity of NO increased in the presence of H_2_O. These results align with the less negative *n*(H_2_O) for NO formation and support the possibility that N_2_O formed at downstream bed positions could arise in part from secondary reduction of NO. Thus, while no promotional effect of H_2_O on N_2_O selectivity is observed at very low conversion, such an effect could emerge at higher conversion due to simultaneous suppression of N_2_ formation and promotion of the secondary N_2_O formation pathway due to higher NO formation in the early stages of the bed. The inhibiting effect of H_2_O on the overall catalyst activity, but a beneficial effect on N_2_O selectivity, is also in agreement with a previous report by Noskov et al.^37^ using a Mn/Bi/Al catalyst, although no mechanistic explanation of this phenomenon was provided.^38^

While *n*(N_2_) and *n*(N_2_O) are also negative for each of the reactions in question, their absolute values are much lower than those of *n*(H_2_O) (Table 1), indicating that they can desorb more readily from the catalyst surface. Consequently, inhibition by N_2_ or N_2_O appears to have little impact on the overall rate of NH_3_ conversion or on product distribution. Interestingly, rather than being negative, *n*(NO) was found to be positive, assuming values of 0.49 and 0.26 for N_2_ and N_2_O formation, respectively. For NO itself, a reaction order could not be determined, as its outlet concentration was consistently lower than the amount cofed with NH_3_ and O_2_, indicating net consumption. This behavior is most likely due to the reduction of NO by NH_3_, a process particularly favored in the presence of O_2_ via an SCR-like pathway. This is also in line with the observed maximum of N_2_O formation and NH_3_ consumption being in the second stage of the catalyst bed (Figure 1d), suggesting that this reaction yields a large amount of secondary N_2_O. However, this also suggests that the results of the kinetic analysis in terms of other reaction orders for NO formation have to be taken with caution, as it is difficult to assess how much NO actually formed via NH_3_ oxidation and how much of it reacted before reaching the reactor outlet. SCR activity of the catalyst will be examined in greater detail in a later section.

### Total Reactant Concentration and Coverage Effects

Given the apparent role of surface coverage by NH_*x*_ species in steering the competition between their recombination and oxidation pathways, we investigated how changes in the inlet concentration of NH_3_ and O_2_ impact selectivity, keeping the 1:1 NH_3_/O_2_ ratio constant (Figure 2a). A modest improvement in N_2_O selectivity could be observed (80% vs 83%) when decreasing the reactant concentrations from 8 down to 2 vol %. This gain was also accompanied by an increase in the NO selectivity. This result is in line with a previous report on NH_3_ oxidation to N_2_O over a Mn/Bi/Al catalyst, where the reduction of the inlet NH_3_ concentration resulted in an increase of both N_2_O and NO selectivity, with the latter increasing by a larger factor.^39^ Further dilution below 2 vol % resulted in a sharp drop in N_2_O selectivity, which was not offset by a corresponding increase in N_2_, but instead by a rise in NO. We can interpret these results in terms of changes in the surface coverage by NH_*x*_ intermediates. Given that NH_3_ oxidation over Mn_SA_/CeO_2_ proceeds with the participation of lattice oxygen of CeO_2_, there is an abundance of oxygen from the lattice of the catalyst, and adsorbed NH_*x*_ species can be rapidly oxidized.^40,41^ At low *p*(NH_3_), their concentration and surface density will be relatively low, and thus the chances of them recombining before getting oxygenated are also lower. However, this also means that upon getting oxygenated, the probability of such species (i.e., HNO) combining to form N_2_O before they get fully dehydrogenated and desorb as NO is also reduced. To corroborate this, Mn_SA_/CeO_2_ was evaluated in a temporal analysis of products (TAP) reactor. Under the transient conditions employed for TAP experiments (low *P*, reactant pulse size in the order of nmol),^42^ we see this effect is even more pronounced, with NO selectivity of 75% being reached. Still, it should be noted that even under these extreme conditions, N_2_ formation could not be completely avoided. These findings demonstrate that variation in inlet composition governs not only the overall conversion but also the relative rates of competing elementary steps. While there is a modest selectivity gain at moderate dilution, under excessively lean conditions, diminished NH_*x*_ surface coverage shifts the reaction toward full oxidation to NO. Maximization of the N_2_O yield therefore requires an optimized feed regime that balances suppression of secondary routes while maintaining surface population necessary for selective N–N and N–O coupling.

**Figure 2:**
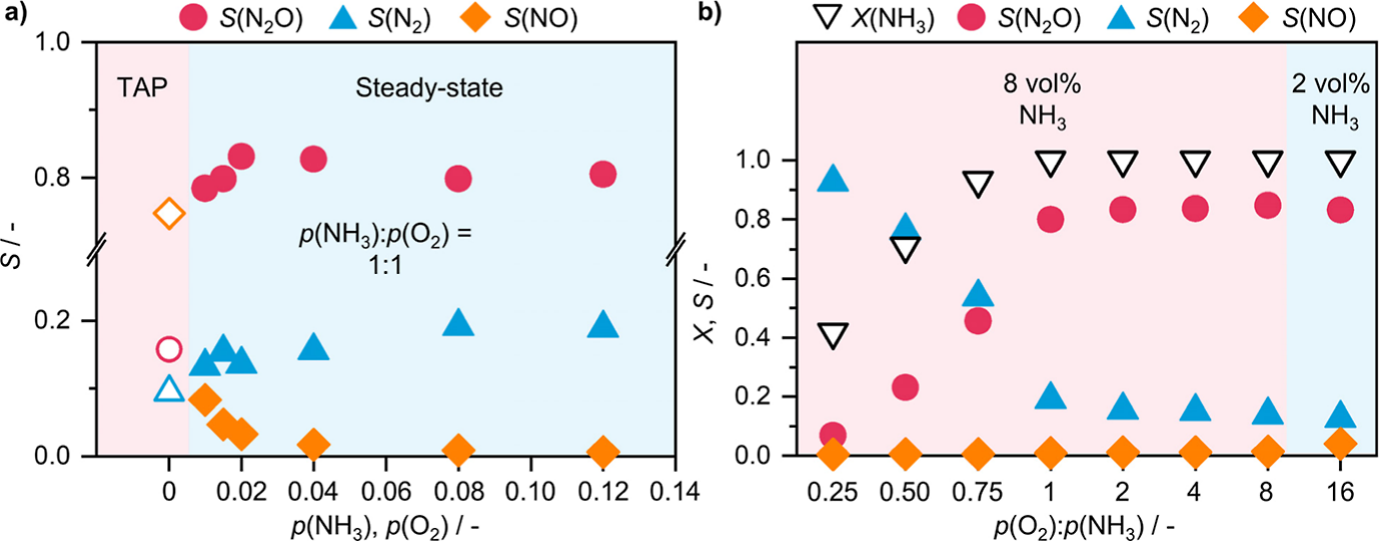
(a) Effect of total reactant partial pressure and (b) ratio of reactant partial pressure on product selectivity of Mn_SA_/CeO_2_ in NH_3_ oxidation. Reaction conditions: *T*_bed_ = 673 K; *m*_cat_ = 0.2 g; GHSV = 15,000 cm^3^ h^–1^ g_cat_^–1^; feed = 2–8 vol % NH_3_, 2–64 vol % O_2_, 4 vol % Ar, He rest; *P* = 1 bar. Reaction conditions: (Steady-state in (a) and (b)) *T*_bed_ = 673 K; *m*_cat_ = 0.2 g; GHSV = 15,000 cm^3^ h^–1^ g_cat_^–1^; feed = 2–8 vol % NH_3_, 2–64 vol % O_2,_ 4 vol % Ar, He rest; *P* = 1 bar; (TAP in a)) *T*_bed_ = 673 K; *m*_cat_ = 0.07 g; feed = 1:1:1 NH_3_/O_2_/He; pulse size = 40 nmol; *P* = 10^–5^ bar.

Additionally, on the basis of kinetic analysis, particularly the effect that partial pressure of O_2_ has on the rate of formation of N_2_O and N_2_, it should follow that supplying O_2_ in a significant excess of NH_3_ should favor N_2_O formation, while excess of NH_3_ should drive selectivity toward N_2_. To test this, the *p*(O_2_)/*p*(NH_3_) ratio was varied over a broad range (0.25–16), and catalytic performance was evaluated at each feed composition. It should be noted that as the objective was to see whether the gap between previously obtained the 81% and 100% N_2_O selectivity can be closed following this approach, the catalytic tests were conducted at low GHSV (Figure 2b). At substoichiometric ratios (*p*(O_2_)/*p*(NH_3_) < 1), N_2_ selectivity increases sharply at the expense of N_2_O, which is consistent with both kinetic arguments and the lack of sufficient oxidant to sustain N_2_O formation. Conversely, providing O_2_ in excess did promote N_2_O selectivity, but only to a limited degree: even at an 8-fold increase in *p*(O_2_), the maximum observed N_2_O selectivity was 85%, only slightly above the 81% baseline. It should also be noted that virtually no difference in NO selectivity was observed, and hence, overoxidation is not the reason for N_2_O selectivity not increasing further. Further increasing the *p*(O_2_)/*p*(NH_3_) ratio to 16, by reducing the inlet NH_3_ concentration, brought no additional benefit. In fact, N_2_O selectivity slightly decreased while NO selectivity rose to 4%, leading to higher overall NO_*x*_ production. This suggests that at very high O_2_ excess, overoxidation begins to play a role, but it still does not account for the persistent 13% of N_2_ formed. This result stands in contrast to the foundational study by Krauss and Neuhaus,^43^ where N_2_O selectivity in NH_3_ oxidation at 573 K over NiO could be dramatically enhanced through the use of excess O_2_ from 25 up to 73%. Similarly, in the context of NH_3_ oxidation to NO, O_2_ was reported to have a strong effect on NO selectivity, capable of driving it up to 100% at sufficient excess.^9^ However, our findings are in line with the work of Noskov et al. using a Mn/Bi/Al catalyst, where only a marginal improvement of 3% in N_2_O selectivity was observed upon an almost 9-fold increase in O_2_ concentration.^37^ The fact that Mn_SA_/CeO_2_ did not reach 100% NO_*x*_ selectivity using feeds with a high O_2_/NH_3_ ratio indicates that kinetically driven N_2_ formation at the very beginning of the catalyst bed could be occurring too fast for additional O_2_ in the stream to have an impact, which would be the case if NH_3_ saturates the catalyst surface before a significant amount of O_2_ can be activated. This interpretation also aligns with the results depicted in Figure 2a, where lowering *p*(NH_3_) even at a stoichiometric feed ratio was beneficial to both NO and N_2_O selectivity. Furthermore, other phenomena, such as side reactions involving primary oxidation products, could be responsible for the residual N_2_ formation and need to be further investigated.

### Secondary Reactions in NH_3_ Oxidation

The kinetic analysis and assessment of segmental rates of NH_3_ oxidation has indicated that while irreversible formation of N_2_ in the very thin upstream layers of the catalyst bed is likely the main reason for N_2_O selectivity losses, other reactions should also be occurring in the downstream layers. In particular, while it appears that N_2_O actually forms via such a reaction, namely, i-SCR of NO, it is also important to consider other reactions that might be taking place. Since N_2_ is too thermodynamically stable to be reoxidized and can be considered a “dead end”, attention is focused on reactions involving N_2_O. These can result in direct losses of N_2_O, namely through N_2_O decomposition into N_2_ and O_2_ (deN_2_O), or N_2_O reduction by NH_3_. Furthermore, N_2_ formation via an i-SCR route is also likely to occur. To evaluate the potential impact of these reactions on the observed product distribution, the reactivity of Mn_SA_/CeO_2_ toward individual reactions was evaluated.

The simplest reaction that might occur is the decomposition of N_2_O into N_2_ and O_2_, according to eq 4. Under a feed of 4 vol % N_2_O, corresponding to the concentration expected at full NH_3_ conversion with complete N_2_O selectivity, Mn_SA_/CeO_2_ exhibited measurable activity, reaching 11% N_2_O conversion at 673 K (Figure S9). However, N_2_O decomposition (deN_2_O) is known to be strongly inhibited by O_2_ and particularly H_2_O.^44^ Indeed, cofeeding H_2_O in the relevant concentration almost completely suppressed activity, delaying the light-off by ∼100 K and eliminating measurable decomposition at 673 K. Thus, under realistic NH_3_ oxidation conditions, deN_2_O is unlikely to contribute significantly to product distribution.

Another reaction that could be taking place is N_2_O reduction by NH_3_, which can yield either N_2_ only (eq 6) or also NO (eq 7). Mn_SA_/CeO_2_ can effectively catalyze this reaction, achieving 70% N_2_O conversion at 673 K, yielding N_2_ with >99% selectivity (Figure 3a). However, it should be noted that under NH_3_ oxidation conditions N_2_O and O_2_, both being oxidants, will be in competition for NH_3_. Therefore, to probe to what extent N_2_O is getting lost via this route, we simulate a scenario where half of the reactants fed under standard conditions (8 vol % NH_3_, 8 vol % O_2_) have been converted into N_2_O (2 vol %) and use this stream composition as the feed. This is then contrasted with a N_2_O-free feed with an equivalent amount of NH_3_ and O_2_, and O_2_ conversion at the reactor outlet is used to evaluate the relative contribution of either oxidant (Figure 3b). At the same total flow of 50 cm^3^ min^–1^, a 2.6% drop in O_2_ conversion in the presence of N_2_O was observed, while full NH_3_ conversion is achieved in either case. Considering the reaction stoichiometry and the fact that NH_3_ oxidation by N_2_O has a dual detrimental effect on N_2_O yield, both directly consuming N_2_O and preventing NH_3_ from being transformed into the desired product, a 2.6% decrease in O_2_ conversion translates into a maximum theoretical loss of 10.4% N_2_O selectivity. While this calculation is based on the assumption that the entirety of the O_2_ that was not consumed in the presence of N_2_O would have been used to convert NH_3_ into N_2_O and therefore likely overestimates the N_2_O loss, it is nevertheless evident that this reaction can diminish the final N_2_O yield. However, upon increasing the total feed flow rate (Figure 3b) and hence reducing the contact time while still maintaining complete NH_3_ conversion, the difference in the amount of O_2_ consumed becomes less significant. This agrees with the results of the kinetic analysis in Table 1, where *n*(N_2_O) was only slightly negative (−0.09) for overall NH_3_ conversion, and hence there was no indication that the rate of NH_3_ conversion was enhanced in the presence of N_2_O. Given that N_2_O is a milder oxidant compared to O_2_ and the extremely short contact times required for kinetic testing, virtually no NH_3_ was oxidized by N_2_O, indicating that the reaction between NH_3_ and N_2_O is much slower than that between NH_3_ and O_2_.

**Figure 3:**
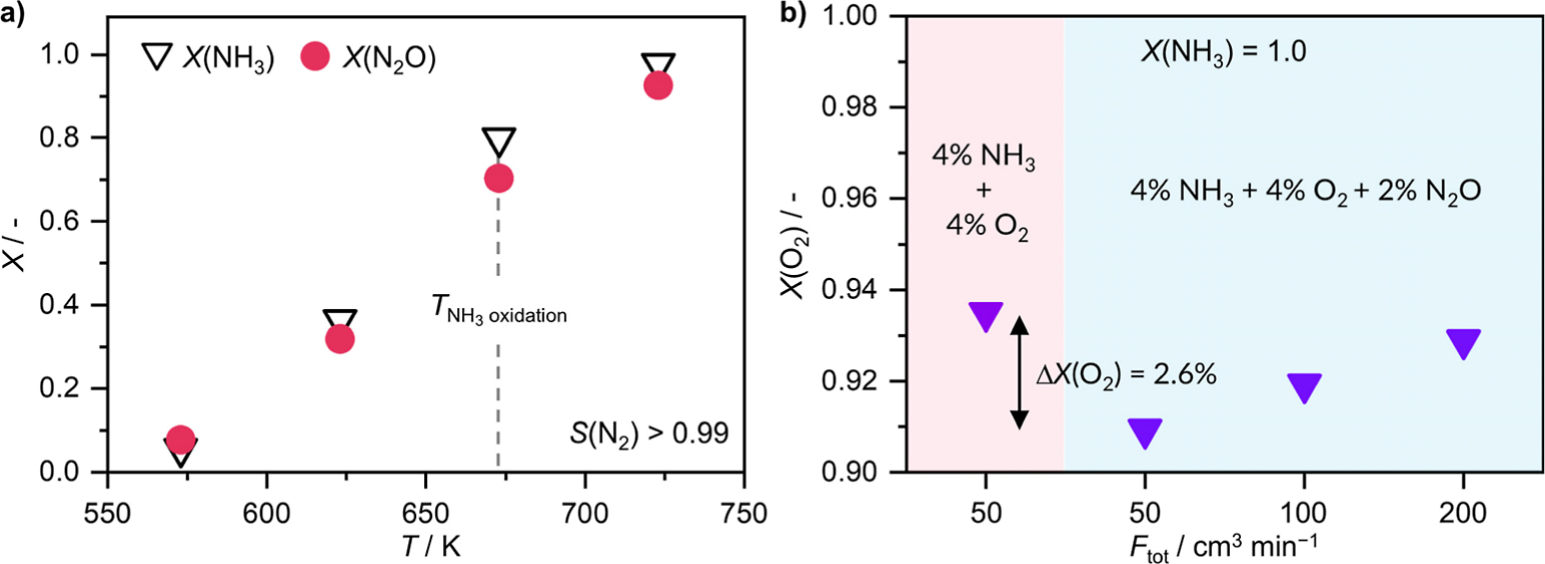
(a) NH_3_ and N_2_O conversion as a function of reaction temperature and (b) O_2_ conversion, in the presence and absence of N_2_O, as a function of total inlet flow rate, of Mn_SA_/CeO_2_ in NH_3_ oxidation. Full conversion of NH_3_ is achieved in all cases in (b). Reaction conditions: *T*_bed_ = (a) 573–773 K or (b) 673 K; *m*_cat_ = 0.2 g; GHSV = 15,000–60,000 cm^3^ h^–1^ g_cat_^–1^; feed = (a) 2.68 vol % NH_3_, 4 vol % N_2_O, 4 vol % Ar, He rest or (b) 4 vol % NH_3_, 4 vol % O_2_, 0–2 vol % N_2_O, 4 vol % Ar, He rest; *P* = 1 bar.

To further probe the potential impact of this reaction, we have also performed kinetic analysis to determine the reaction orders with respect to NH_3_ and N_2_O (Figure S10). It should be noted that much milder reaction conditions than in NH_3_ oxidation by O_2_ were required for reactant conversion to be sufficiently low to ensure differential reactor operation (*X* < 20%), further supporting the interpretation that O_2_ is a more potent oxidant for NH_3_ oxidation compared to N_2_O. The analysis revealed *n*(NH_3_) = 0.66 and *n*(N_2_O) = 1.59. The large value of *n*(N_2_O) is consistent with the fact that, as opposed to O_2_, N_2_O is a mono-oxygen donor, and thus double the equivalent of N_2_O is required to generate the same number of O* species. Namely, the stoichiometric requirement is 1.5 mol N_2_O to convert one mole of NH_3_ (eq 6, in view of observed >99% selectivity to N_2_). Accordingly, as most steps along the way to N_2_ involve two species (eqs 26–32), whose concentrations will be dependent on O* coverage, which in turn will depend strongly on *p*(N_2_O), it is reasonable to anticipate *n*(N_2_O) exceeding unity. The large value of *n*(N_2_O) and relatively lower value of *n*(NH_3_) suggest that the reaction might start occurring in the intermediate segments of the catalyst bed (Figure 1c,d, segments 2–4), where some N_2_O has already formed but NH_3_ has not yet been fully consumed. However, given that we observe virtually no N_2_ formation in the downstream 90% of the catalyst bed, we can exclude the possibility that N_2_O reduction by residual NH_3_ is occurring to any appreciable extent, which further suggests that the intrinsic rate of the reaction is too low to significantly impact the final product distribution.

### Secondary N_2_ and N_2_O Formation via Internal SCR

Kinetic analysis of NH_3_ oxidation revealed a positive *n*(NO) for the formation of both N_2_O and N_2_ (Table 1). Furthermore, we observed the rate of N_2_O formation and NH_3_ consumption passing a maximum when assessing segmental reaction rates (Figure 1d), pointing to the generation of N_2_O via a secondary pathway. This gives a strong indication that Mn_SA_/CeO_2_ can catalyze SCR, proceeding according to eqs 8–10. To unequivocally confirm this, a test with stoichiometric feed composition for N_2_O formation (4 vol % NH_3_, 3 vol % O_2_, 4 vol % NO) was performed (Figure 4). This feed contains an equivalent number of N and O atoms as the standard feed under NH_3_ oxidation conditions (8 vol % NH_3_, 8 vol % O_2_). Accordingly, it can serve as a proxy for the scenario in which half of NH_3_ in the feed gets initially oxidized to NO. Under these conditions, NO conversion is already high at 473 K and exceeds 90% by 573 K, confirming that NH_3_ is not oxidized solely by O_2_ but also by NO through SCR pathways. It should also be noted that no NO_2_ was detected at the reactor outlet even at low temperature, suggesting that even if it forms, it gets consumed to oxidize NH_3_ in a fast-SCR reaction. At higher temperatures (*T* > 623 K), however, NO conversion starts to gradually decrease, consistent with a growing contribution of direct NH_3_ oxidation to the overall reactivity. It is also notable that under SCR conditions the catalyst still generates a substantial amount of N_2_O. Maximum N_2_O selectivity of 69% could be attained at 623 K, which nevertheless falls below the optimum temperature for N_2_O selectivity (673 K) and below the maximum N_2_O selectivity attainable under NH_3_ oxidation conditions (81%). In fact, while at 673 K N_2_O is still the major product, the selectivity falls to 58%, which also coincides with the drop in NO conversion. This further suggests that also under NH_3_ oxidation conditions a part of observed N_2_O originates via an i-SCR pathway.

**Figure 4:**
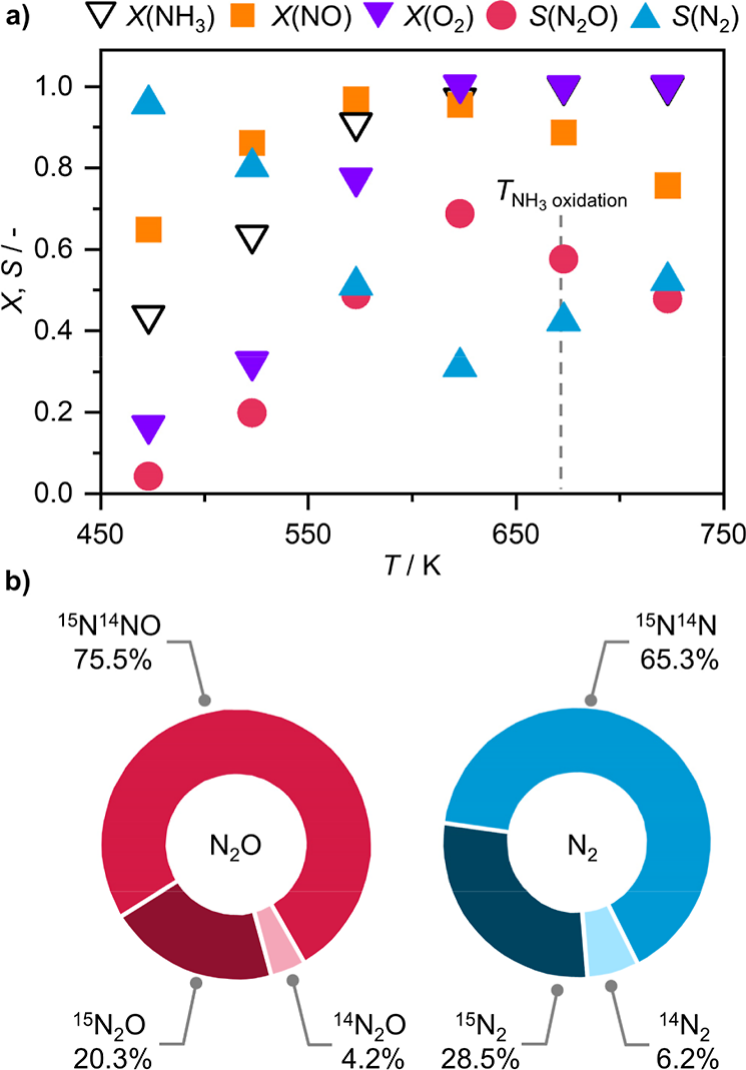
(a) Reactant conversion and product selectivity of Mn_SA_/CeO_2_ in SCR as a function of reaction temperature and (b) distribution of N_2_O and N_2_ isotopologues when isotopically labeled ^15^NH_3_ is used. Reaction conditions: *T*_bed_ = (a) 473–773 K or (b) 673 K; *m*_cat_ = 0.2 g; GHSV = 15,000 cm^3^ h^–1^ g_cat_^–1^; feed = 4 vol % (a) ^14^NH_3_ or (b) ^15^NH_3_, 4 vol % NO, 3 vol %, 4 vol % Ar, He rest; *P* = 1 bar.

To disentangle the contribution of SCR and NH_3_ oxidation pathways to N_2_O and N_2_ formation in Figure 4a and hence to estimate the potential impact of i-SCR during NH_3_ oxidation, tracing experiments with isotopically labeled ^15^NH_3_ were performed at 673 K (Figure 4b). The dominant isotopologue of N_2_O was ^15^N^14^NO, which is consistent with the stoichiometry and mechanism of the SCR reaction,^21^ with NH_3_ and NO each contributing one N atom to the final product. Similarly, ^15^N^14^N is the most abundant isotopologue of N_2_, again pointing to the involvement of NO. However, we also observed a significant amount of ^15^N_2_O, making up 20% of the total amount of N_2_O formed. While it is not possible to unambiguously determine the fraction of ^15^N_2_O formed through direct oxidation of ^15^NH_3_ to ^15^N_2_O, or through an i-SCR mechanism involving the reduction of ^15^NO, formed through oxidation of ^15^NH_3_ to ^15^NO, the substantial quantity of ^15^N^14^NO (Figure 4b), which should be formed through the latter mechanism, indicates that ^15^N_2_O is also predominantly formed in this way.

Similarly, nearly one-third of the N_2_ stream was ^15^N_2_, which, at least in part, could be attributed to O_2_-mediated NH_3_ oxidation. Notably, we also see a small amount of ^14^N_2_O in the product stream. Given the high isotopic purity of ^15^NH_3_ used for these experiments, this cannot be attributed solely to ^14^NH_3_ impurities and instead indicates that minor quantities of N_2_O can also form through dimerization of NO or through recombination of adsorbed NO with a deoxygenated NO fragment. Control experiments with NO-only feeds confirmed that while some N_2_O forms, the amount is negligible compared to mixed feeds. This suggests that the contribution of NO dimerization under the reaction conditions is practically negligible, and the ^14^N fragment had to originate from N–O bond scission of NO, facilitated by ^15^NH_3_ acting as a reducing agent. These findings clearly demonstrate that several pathways for the formation of both N_2_O and N_2_ are in play under SCR conditions. Based on the higher N_2_O selectivity observed under SCR conditions (Figure 4a), it is highly likely that the i-SCR mechanism contributes to the formation of both N_2_O and, to a lesser extent, N_2_ under NH_3_ oxidation conditions.

Given that Mn–CeO_2_ systems are already known to exhibit SCR activity,^31,45^ it is important to establish whether this behavior extends to other leading catalysts for selective N_2_O synthesis, namely, Au_NP_/CeO_2_ and CrCeO_*x*_. Accordingly, their reactivity in SCR was evaluated (Figure S11). Both catalysts proved highly active in SCR, with Au_NP_/CeO_2_ achieving full NO conversion already at 523 K. In fact, while CrCeO_*x*_ exhibited behavior highly reminiscent of Mn_SA_/CeO_2_, with maximum N_2_O selectivity in the 60–70% range attained at 623 K, Au_NP_/CeO_2_ could reach N_2_O selectivity of 79% at 573 K, which also coincides with the optimal temperature for N_2_O formation under NH_3_ oxidation conditions. Performing isotopic labeling experiments with ^15^NH_3_ on these systems further confirmed clear parallels in product distribution between Mn_SA_/CeO_2_ and CrCeO_*x*_. Interestingly, however, this experiment revealed a distinctive feature of Au_NP_/CeO_2_. It yielded an even higher fraction of ^15^N^14^N and ^15^N^14^NO, which clearly originated through an SCR-like mechanism, while also being the most selective to the N_2_O system under both SCR and NH_3_ oxidation conditions. This provides additional evidence that not only is the i-SCR mechanism in NH_3_ oxidation likely to be contributing to product formation but also that the tendency to form N_2_O via i-SCR could be among the key factors leading to the higher N_2_O selectivity of Au_NP_/CeO_2_ under NH_3_ oxidation conditions.

### Reaction Engineering Strategies

The assessment of secondary reactions has revealed that i-SCR activity of the catalyst could be an important factor determining the final product distribution and is therefore an additional aspect to consider when designing NH_3_ oxidation catalysts. Reaction engineering can often be a more practical approach to drive N_2_O selectivity up. We have observed that H_2_O cofeeding, reducing reactant concentration and supplying excess of O_2_ could all yield marginal improvements. However, while the latter is of phenomenological interest, it is not a practical method, as downstream removal of unreacted O_2_ is challenging and costly. On the other hand, given the high activity of the catalyst, reducing inlet concentrations is not problematic in terms of N_2_O productivity, and H_2_O cofeeding is also technically straightforward. Thus, we investigated whether the two methods can be complementary and allow us to reach a higher N_2_O selectivity (Figure 5). When reducing inlet concentrations of NH_3_ and O_2_ down to 2 vol % and cofeeding 60 vol % of H_2_O, compared to inlet concentrations of 8 vol % with no H_2_O in the feed, an 11% decrease in N_2_ selectivity could be attained, while N_2_O selectivity increased from 81 to 90%, showing successful suppression of undesired N_2_ formation and driving the overall NO_*x*_ (N_2_O + NO) selectivity to 94%. This improvement in N_2_O selectivity is more significant than what had been previously achieved by changing the composition and active site nanostructure of the leading CeO_2_-based catalysts, highlighting the underutilized power of reaction engineering. Still, it should be acknowledged that cofeeding of steam at 60 vol % is not realistic from a technical point of view in an industrial setting. This experiment serves to demonstrate the impact of our kinetic analysis for enhancing N_2_O selectivity without the need to modify the catalyst rather than to propose an industrially optimized operating strategy. Indeed, the latter will necessitate that both the reaction and catalyst engineering aspects be developed, optimized, and utilized in tandem, leaving ample room for further investigations.

**Figure 5:**
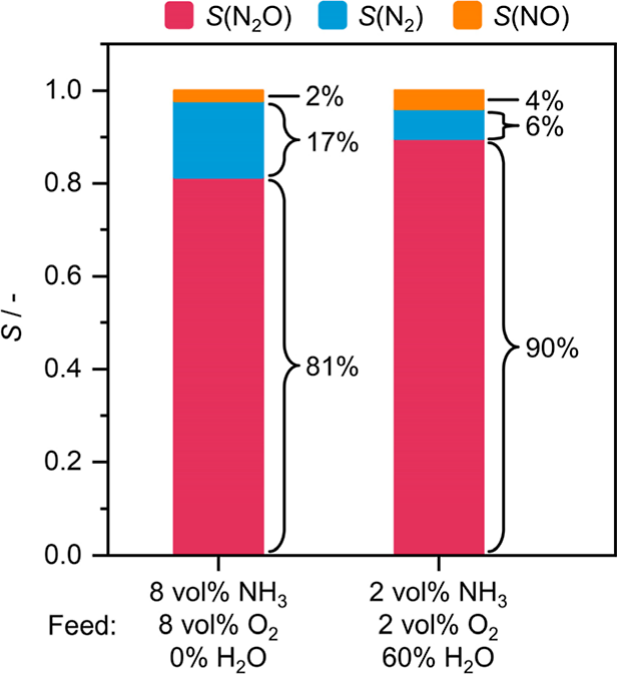
Combined effect of H_2_O cofeeding and reducing reactant partial pressure on the product selectivity of Mn_SA_/CeO_2_ in NH_3_ oxidation. Reaction conditions: *T*_bed_ = 673 K; *m*_cat_ = 0.2 g; GHSV = 15,000 cm^3^ h^–1^ g_cat_^–1^; feed = 2–8 vol % NH_3_, 2–8 vol % O_2,_ 0–60 vol % H_2_O, 4 vol % Ar, He rest; *P* = 1 bar.

## Conclusion

In this work, a comprehensive kinetic study also in a spatially resolved manner, was conducted, aimed at identifying selectivity gaps in NH_3_ oxidation catalysts for N_2_O synthesis and using Mn_SA_/CeO_2_ as a representative benchmark (Figure 6). It revealed that N_2_ formation is strongly favored at the early stages of the catalyst bed due to its strong dependence on NH_3_ partial pressure and hence surface coverage by N-containing intermediates. However, due to strong inhibition of this reaction by H_2_O formed during the reaction and lower stoichiometric requirements of N_2_ formation for O_2_, as the NH_3_ conversion increases and the gas composition shifts toward O_2_- and H_2_O-rich conditions, N_2_O becomes the dominant product in downstream catalyst layers. Furthermore, changing the partial pressure of reactants was shown to be an effective method of tuning surface coverage by N-containing intermediates and enhancing N_2_O selectivity, albeit one that has to be balanced against excessive dilution, which results in NO formation being favored. By combining moderate dilution with H_2_O cofeeding, N_2_O selectivity of the catalyst could be improved from 81% to 90%, suggesting that underlying reasons for N_2_O selectivity losses under standard operating conditions were correctly identified. Secondary reactions involving primary oxidation products were also evaluated: while direct N_2_O decomposition and N_2_O reduction by NH_3_ were found to have little to no impact, the reduction of in situ formed NO by NH_3_ was found to have a significant effect on product distribution. Isotopic labeling experiments revealed that the latter serves as an additional pathway for the formation of N_2_O and, to a lesser extent, of N_2_. The contribution of this reaction to N_2_O formation increases along the catalyst bed. In fact, SCR activity was demonstrated to be a general feature of CeO_2_-based catalysts for NH_3_ oxidation to N_2_O, which significantly contributes to the final N_2_O yield and therefore represents an additional aspect of the catalyst design that can be tuned to enhance selectivity. Importantly, these insights were derived primarily from steady-state experiments and kinetic analyses. While such approaches have become less commonly emphasized in recent years, this study highlights their power in disentangling complex reaction networks and revealing overlooked aspects of catalyst function. We therefore argue that rigorous kinetic analysis should remain central in future investigations of NH_3_ oxidation to N_2_O, and, more broadly, in the rational design of heterogeneous catalysts.

**Figure 6:**
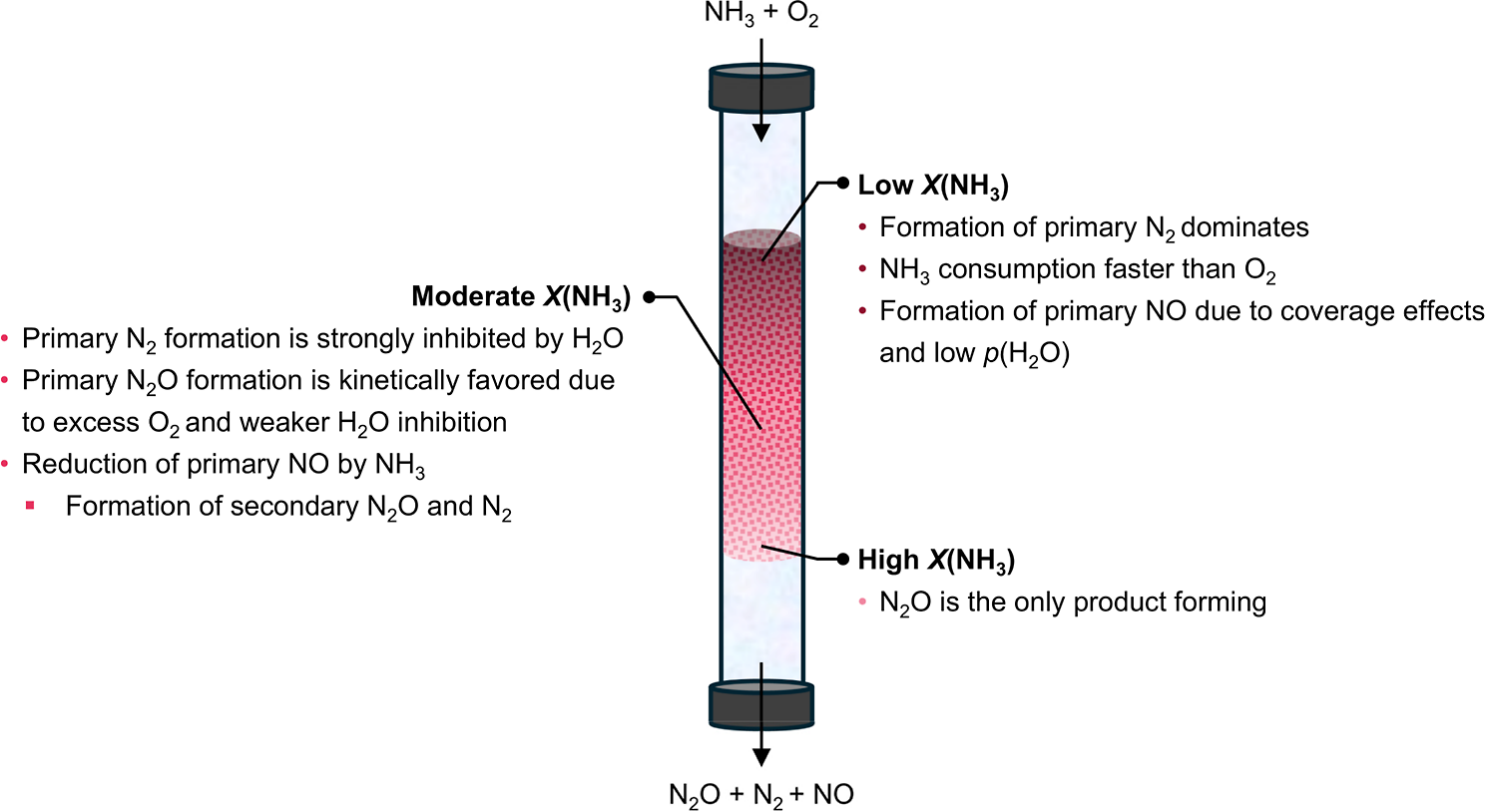
Schematic summary of reactions occurring at different stages of the catalytic bed during NH_3_ oxidation, contributing to the eventual product distribution.

## Supplementary Material



## Data Availability

The experimental data presented in the main figures of the manuscript are publicly available through Zenodo (10.5281/zenodo.17165021).

## Notes

Mass and heat transfer limitations during catalytic tests, scheme of the setup for catalytic tests, catalytic data as a function of catalyst particle size and flow rate, influence of dilution by silicon carbide on catalytic results, catalytic data at different temperatures and partial pressures of reactants and products to determine apparent activation energies and reaction orders, influence of water cofeeding on product selectivity, catalytic activity in N_2_O decomposition and N_2_O-mediated NH_3_ oxidation, and performance of Au and Cr-based catalysts in NO reduction by NH_3_, including the production distribution using isotopically labeled ammonia (PDF)
