# Supplementary material for: Origins of N2O Selectivity Limits in Catalyzed Ammonia Oxidation
Source: ACS Catal. 2026 Feb 4;16(4):3231–42. doi: 10.1021/acscatal.5c07065 (PMC12939828; doi:10.1021/acscatal.5c07065)
Supplement: Supplementary file 1 [file cs5c07065_si_001.pdf]

Supporting Information

Origins of N_2O Selectivity Limits in Catalyzed Ammonia Oxidation

Ivan Surin,[1] *Evgenii V. Kondratenko*[2]* *and Javier Pérez-Ramírez*[1,3]*

[1] Institute of Chemical and Bioengineering, Department of Chemistry and Applied Biosciences, ETH Zurich, Vladimir-Prelog-Weg 1, 8093 Zurich, Switzerland.

[2] Advanced Methods for Applied Catalysis, Leibniz-Institut für Katalyse, Albert Einstein-Str. 29a, 18059 Rostock, Germany.

[3] NCCR Catalysis, 8093 Zurich, Switzerland.

* Corresponding authors. E-mails: evgenii.kondratenko@catalysis.de; jpr@chem.ethz.ch

Supporting Tables.

Table S1. Results for mass and heat transfer limitation criteria for Mn_{SA}/CeO_2 at distinct conditions.

Catalyst	$r_{v,obs}$[a] / mol s^{-1} m^{-3}	γ[b] / -	Ca[c] / -	β_e[d] / -	$\lvert\gamma \cdot \beta_e\ Ca\rvert$[e] / -	Φ[f] / -	β_i[g] / -	$\lvert\gamma \cdot \beta_i\ \Phi\rvert$[h] / -
Mn_{SA}/CeO_2	3.07	17.3	0.004	0.40	0.001	$7 \cdot 10^{-4}$	0.03	$4 \cdot 10^{-4}$
Mn_{SA}/CeO_2	148	17.3	$2 \cdot 10^{-4}$	0.27	0.02	0.033	0.03	0.01

[a] Observed reaction rate. Reaction conditions: $T = 673$ K; $m_{cat} = 0.2$ or 0.001 g; $GHSV = 15'000$ or 9'000'000 $cm^3\ h^{-1}\ g_{cat}^{-1}$; Feed = 8 vol% NH_3, 8 vol% O_2, 4 vol% Ar, 80 vol% He; $P = 1$ bar. [b] Dimensionless activation energy. [c] Carberry criterion. [d] External Prater number. [e] Non-isothermal criterion for absence of external mass and heat transfer limitations. [f] Weisz-Prater criterion. [g] Internal Prater number. [h] Non-isothermal criterion for absence of internal mass and heat transfer limitations.

Supporting Figures.

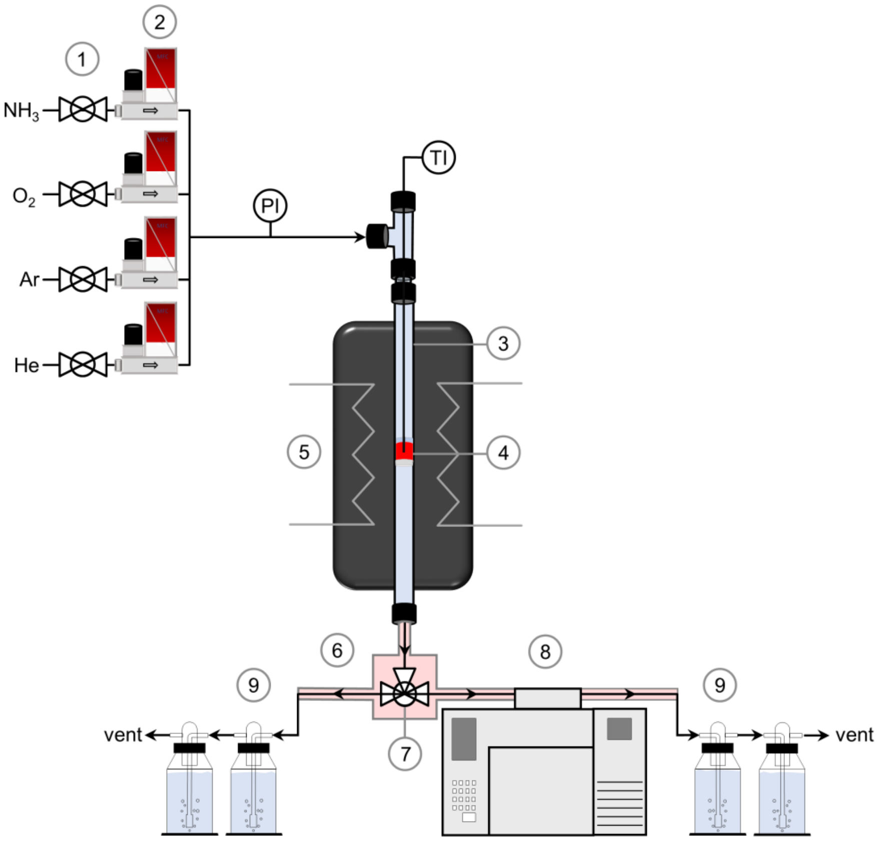

Figure S1. Diagram of the laboratory set-up used for ammonia oxidation. 1: two-way on/off valves, 2: mass flow controllers, 3: quartz reactor, 4: catalyst bed, 5: oven, 6: heat tracing (red background), 7: three-way sampling valve, 8: gas chromatograph coupled to a mass spectrometer (GC-MS), 9: H_2O and H_2SO_4 scrubbers, PI: pressure indicator, and TI: temperature indicator.

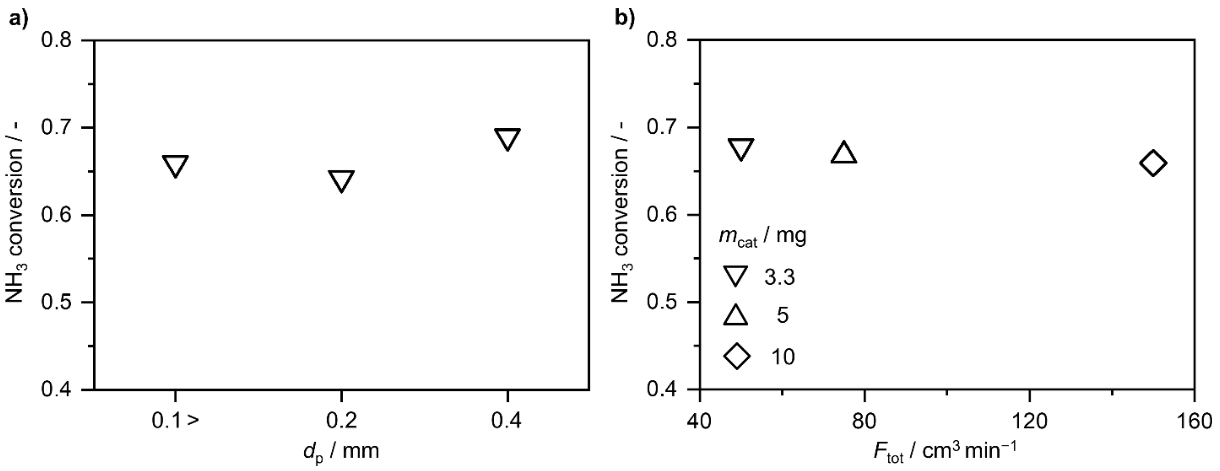

Figure S2. NH_3 conversion of Mn_{SA}/CeO_2 in NH_3 oxidation as a function of **a)** catalyst particle size and **b)** total volumetric flow rate at a fixed *GHSV*. Reaction conditions: T_{bed} = 673 K; m_{cat} = 0.0033-0.01 g; *GHSV* = 900'000 $cm^3\,h^{-1}\,g_{cat}^{-1}$; Feed = 8 vol% NH_3, 8 vol% O_2, 4 vol% Ar, He rest; *P* = 1 bar.

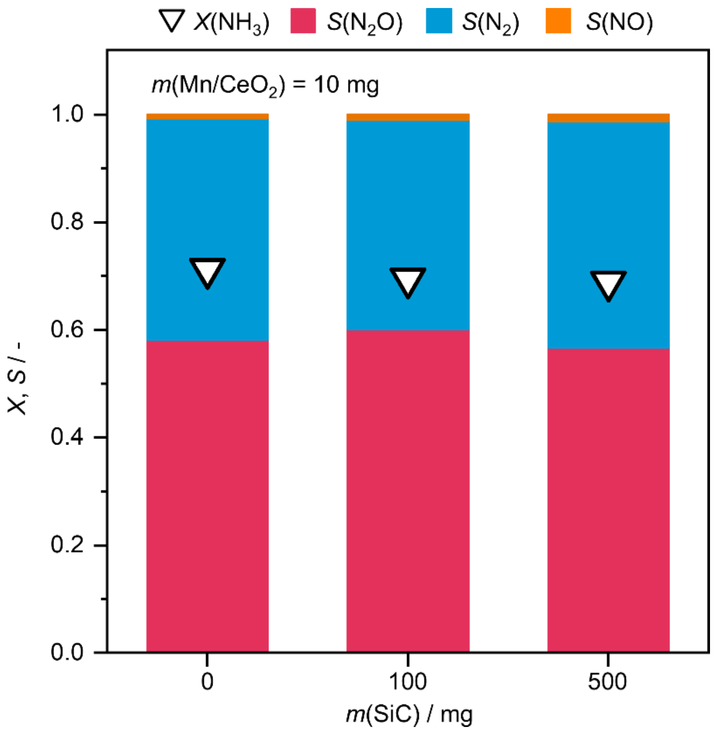

Figure S3. NH_3 conversion and product selectivity of Mn_{SA}/CeO_2 in NH_3 oxidation as a function of the mass of SiC used to dilute the catalyst bed. Reaction conditions: $T_{bed} = 673$ K; $m_{cat} = 0.01$ g; m(SiC) = 0-0.5 g; $GHSV = 600'000$ $cm^3\ h^{-1}\ g_{cat}^{-1}$; Feed = 8 vol% NH_3, 8 vol% O_2, 4 vol% Ar, He rest; $P = 1$ bar.

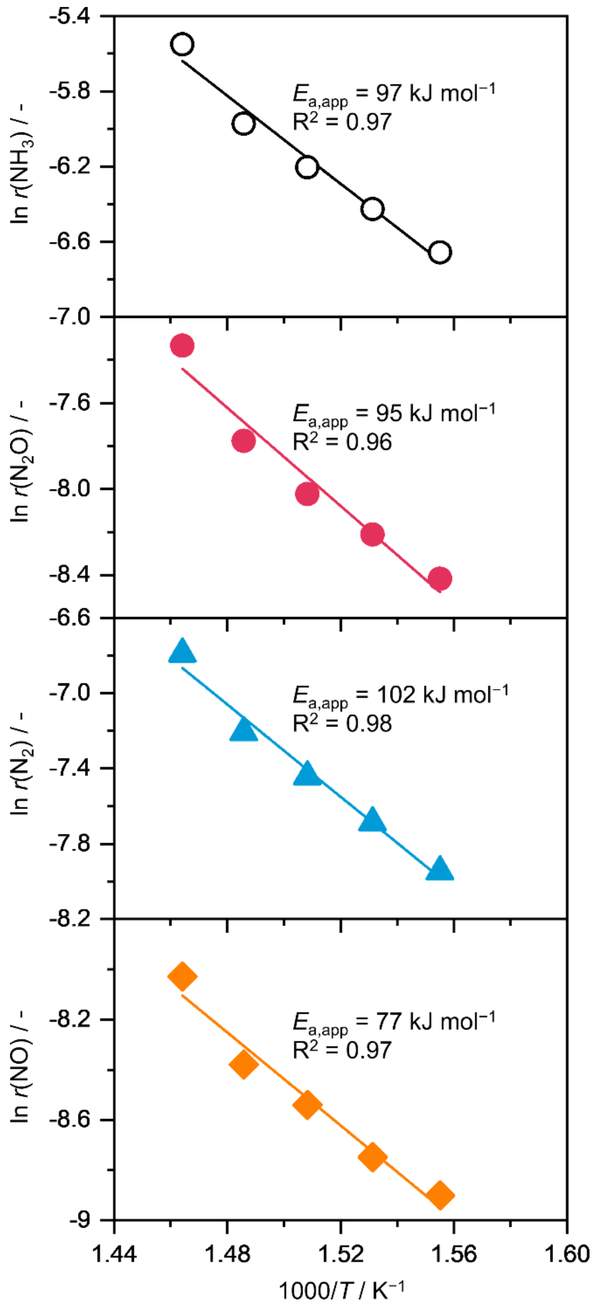

Figure S4. Rate of NH_3 consumption and product formation in NH_3 oxidation as a function of reaction temperature. The corresponding apparent activation energies, $E_{a,app}$, and R^2 values of linear fits are indicated in each panel. Reaction conditions: T_{bed} = 653-683 K; m_{cat} = 0.001 g; *GHSV* = 6'000'000 cm^3 h^{-1} g_{cat}^{-1}; Feed = 8 vol% NH_3, 8 vol% O_2, 4 vol% Ar, He rest; P = 1 bar.

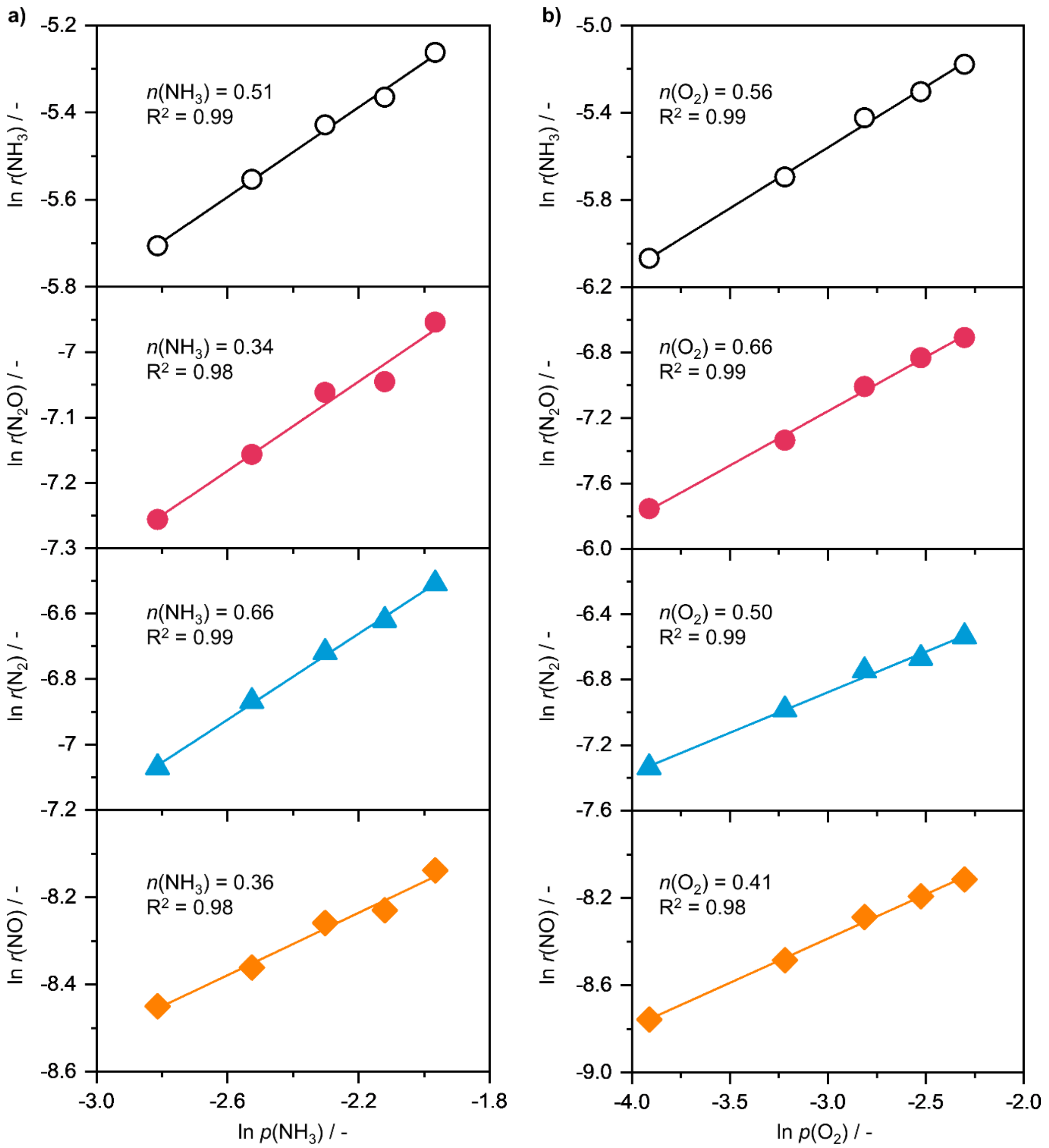

Figure S5. Rate of NH_3 consumption and product formation in NH_3 oxidation as a function of partial pressure of **a)** NH_3 and **b)** O_2. The corresponding reaction orders and R^2 values of linear fits are indicated in each panel. Reaction conditions: $T_{bed} = 673$ K; $m_{cat} = 0.001$ g; $GHSV = 6'000'000$ $cm^3\ h^{-1}\ g_{cat}^{-1}$; Feed = **a)** 6-14 vol% NH_3, 8 vol% O_2, 4 vol% Ar, He rest; **b)** 8 vol% NH_3, 2-10 vol% O_2, 4 vol% Ar, He rest; $P = 1$ bar.

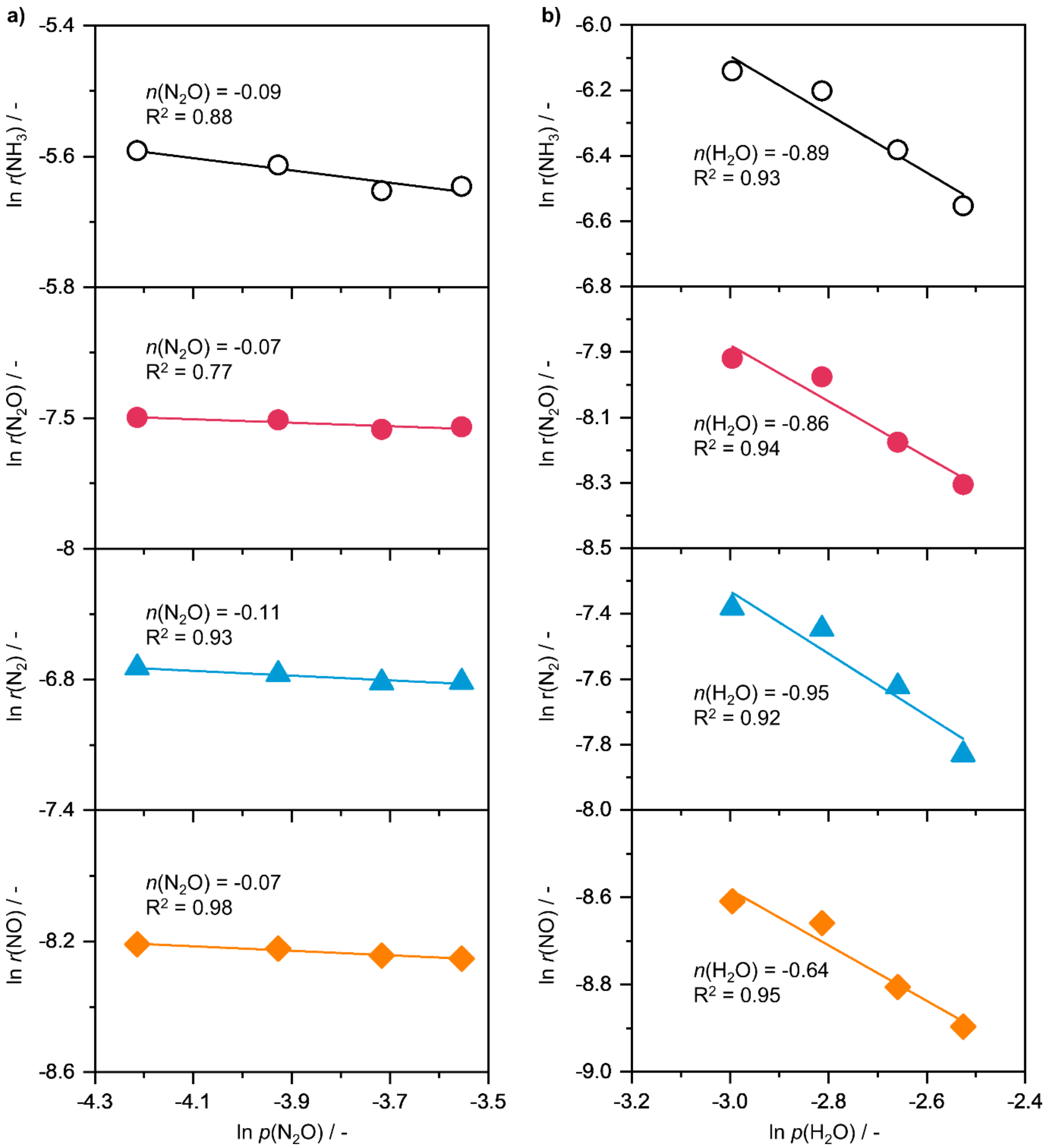

Figure S6. Rate of NH_3 consumption and product formation in NH_3 oxidation as a function of partial pressure of **a)** N_2O and **b)** H_2O. The corresponding reaction orders and R^2 values of linear fits are indicated in each panel. Reaction conditions: $T_{bed} = 673$ K; $m_{cat} = 0.001$ g; *GHSV* = 6'000'000 $cm^3\ h^{-1}\ g_{cat}^{-1}$; Feed = **a)** 8 vol% NH_3, 8 vol% O_2, 0.5-3 vol% N_2O, 4 vol% Ar, He rest; **b)** 8 vol% NH_3, 8 vol% O_2, 5-8 vol% H_2O, 4 vol% Ar, He rest; $P = 1$ bar.

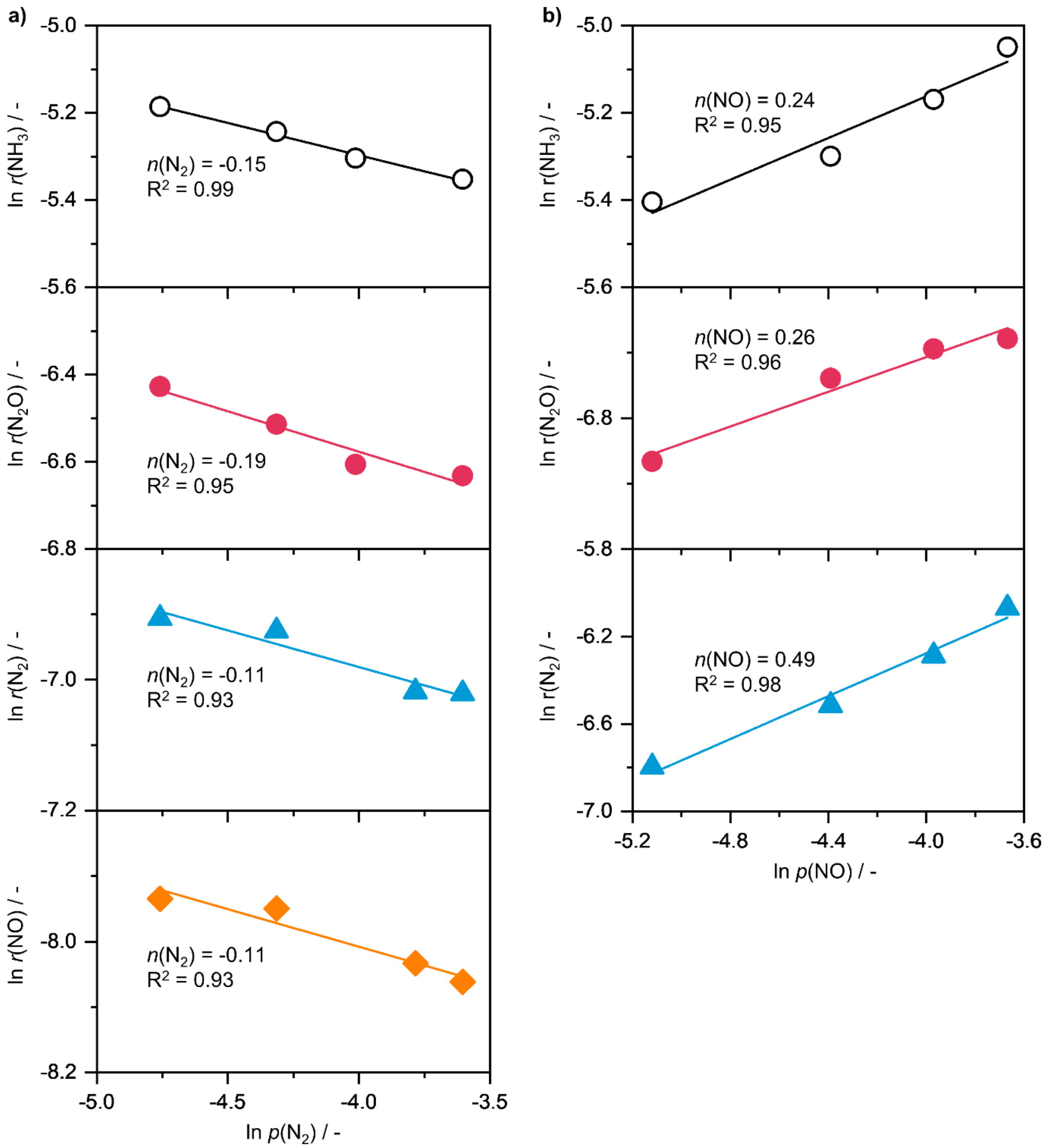

Figure S7. Rate of NH_3 consumption and product formation in NH_3 oxidation as a function of partial pressure of **a)** N_2 and **b)** NO. The corresponding reaction orders and R^2 values of linear fits are indicated in each panel. Reaction conditions: T_{bed} = 673 K; m_{cat} = 0.001 g; *GHSV* = 6'000'000 $cm^3\,h^{-1}\,g_{cat}^{-1}$; Feed = **a)** 8 vol% NH_3, 8 vol% O_2, 1-3 vol% N_2, 4 vol% Ar, He rest; **b)** 8 vol% NH_3, 8 vol% O_2, 0.5-2 vol% NO, 4 vol% Ar, He rest; P = 1 bar.

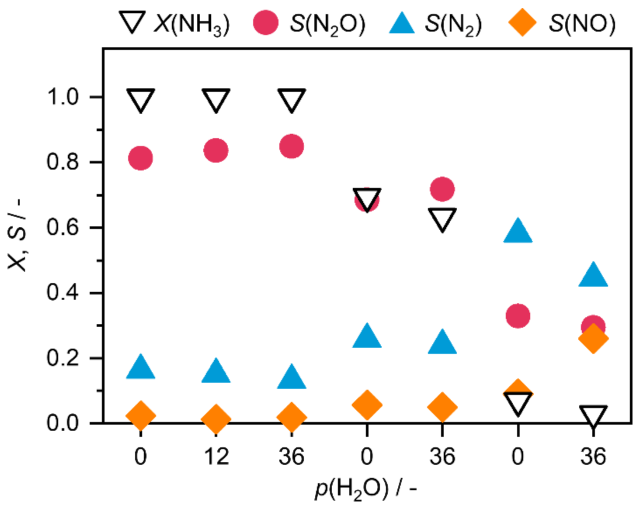

Figure S8. The effect of H_2O co-feeding on product selectivity of Mn_{SA}/CeO_2 in NH_3 oxidation. Reaction conditions: Reaction conditions: T_{bed} = 673 K; m_{cat} = 0.001-0.2 g; *GHSV* = 15'000-9'000'000 $cm^3\ h^{-1}\ g_{cat}^{-1}$; Feed = 8 vol% NH_3, 8 vol% O_2, 0-36 vol% H_2O, 4 vol% Ar, He rest; *P* = 1 bar.

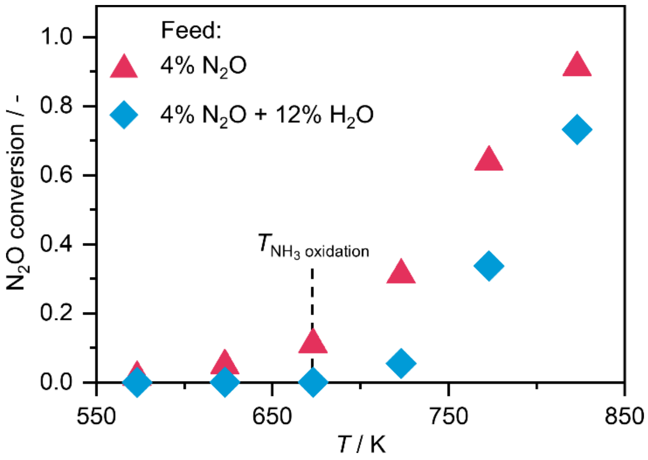

Figure S9. N_2O conversion of Mn_{SA}/CeO_2 in N_2O decomposition as a function of reaction temperature. Reaction conditions: T_{bed} = 573-823 K; m_{cat} = 0.2 g; *GHSV* = 15'000 $cm^3\ h^{-1}\ g_{cat}^{-1}$; Feed = 4 vol% N_2O, 0-12 vol% H_2O, 4 vol% Ar, He rest; P = 1 bar.

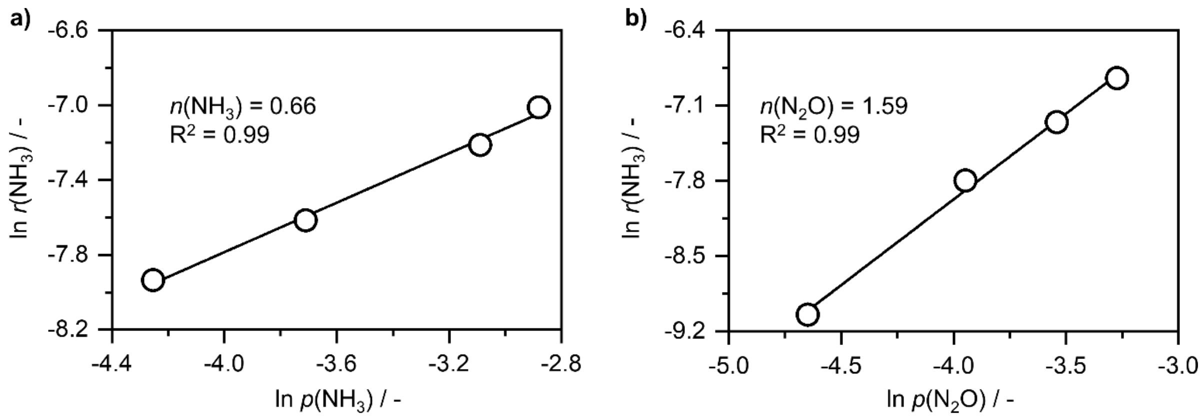

Figure S10. Rate of NH_3 consumption during NH_3 oxidation by N_2O as a function of partial pressure of **a)** NH_3 and **b)** N_2O. The corresponding reaction orders and R^2 values of linear fits are indicated in each panel. Reaction conditions: $T_{bed} = 673$ K; $m_{cat} = 0.01$ g; $GHSV = 600'000$ $cm^3\,h^{-1}\,g_{cat}^{-1}$; Feed = **a)** 2-6 vol% NH_3, 2 vol% N_2O, 4 vol% Ar, He rest; **b)** 4 vol% NH_3, 1-4 vol% N_2O, 4 vol% Ar, He rest; $P = 1$ bar.

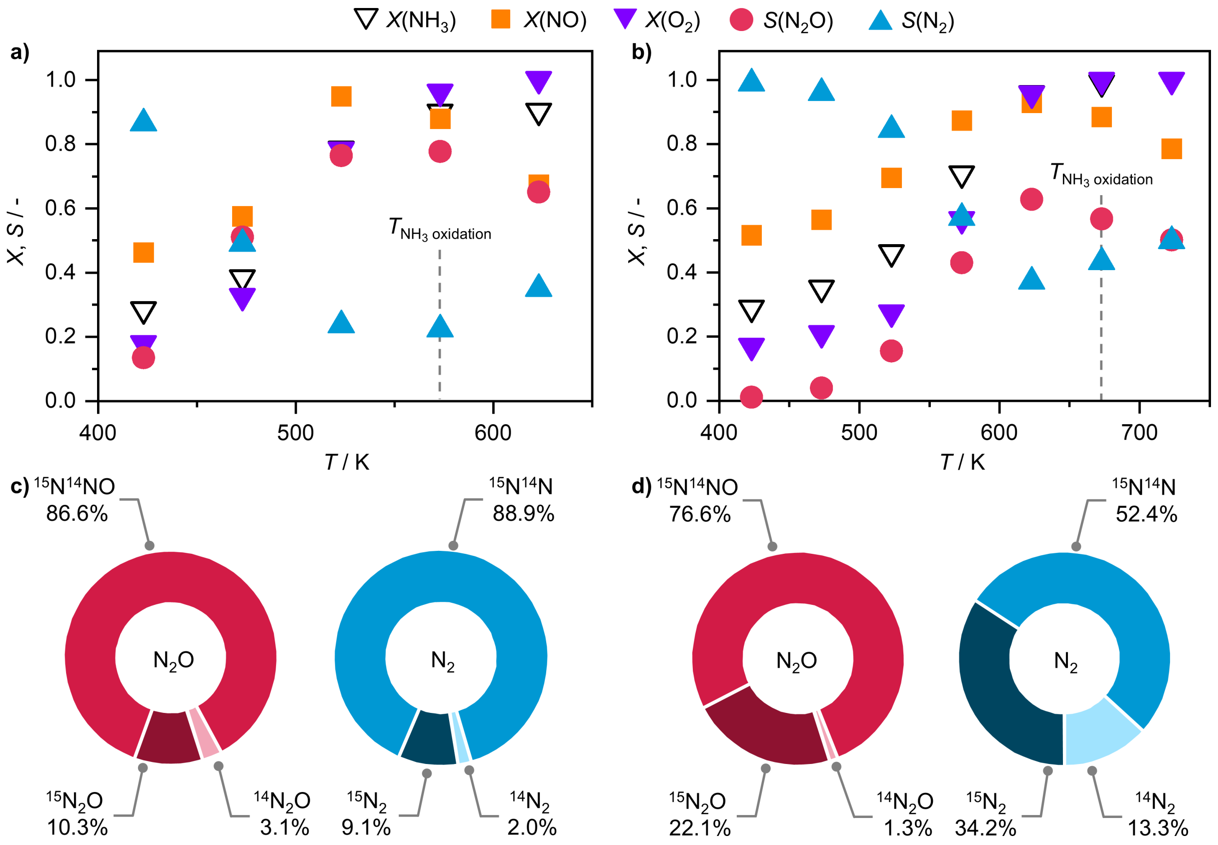

Figure S11. Reactant conversion and product selectivity of reference **a)** Au_{NP}/CeO_2 and **b)** $CrCeO_x$ catalysts in SCR as a function of reaction temperature, and **c,d)** corresponding distributions of N_2O and N_2 isotopologues when isotopically labelled $^{15}NH_3$ is used at 673 K. Reaction conditions: T_{bed} = **a, b)** 473-773 K or **c, d)** 673 K; m_{cat} = 0.2 g; $GHSV$ = 15'000 $cm^3\,h^{-1}\,g_{cat}^{-1}$; Feed = 4 vol% **a, b)** $^{14}NH_3$ or **c, d)** $^{15}NH_3$, 4 vol% NO, 3 vol%, 4 vol% Ar, He rest; P = 1 bar.
